# EGFR and PI3K Signalling Pathways as Promising Targets on Circulating Tumour Cells from Patients with Metastatic Gastric Adenocarcinoma

**DOI:** 10.3390/ijms25105565

**Published:** 2024-05-20

**Authors:** Ann-Katrin Piper, Chelsea Penney, Jacqueline Holliday, Gary Tincknell, Yafeng Ma, Sarbar Napaki, Klaus Pantel, Daniel Brungs, Marie Ranson

**Affiliations:** 1 School of Chemistry and Molecular Bioscience, University of Wollongong, Wollongong, NSW 2522, Australiamranson@uow.edu.au (M.R.); 2 Molecular Horizons, University of Wollongong, Wollongong, NSW 2522, Australia; 3 Illawarra Cancer Care Centre, Wollongong Hospital, Wollongong, NSW 2500, Australia; 4 Centre for Circulating Tumour Cell Diagnostics & Research at the Ingham Institute for Applied Medical Research, South-Western Clinical School, University of New South Wales, Liverpool, NSW 2170, Australia; 5 Graduate School of Medicine, University of Wollongong, Wollongong, NSW 2522, Australia; 6 Department of Pathology, Wollongong Hospital, Wollongong, NSW 2500, Australia; 7 Institute for Tumor Biology, University Cancer Center Hamburg, University Medical Center Hamburg-Eppendorf, 20246 Hamburg, Germany

**Keywords:** circulating tumour cells, gastric cancer, c-Met, PI3K/Akt, EGFR, cell signalling, 3D cell culture, organotypics, PIK-75, alpelisib, gefitinib

## Abstract

The prognosis for metastatic gastric adenocarcinoma (mGAC) remains poor. Gene alterations in receptor tyrosine kinases (RTKs) such as epidermal growth factor receptor *(EGFR)* and their downstream effectors including catalytic subunit alpha of the phosphatidylinositol 3-kinase *(PIK3CA)* are common in mGAC. Targeted RTK and phosphatidylinositol-3-kinase (PI3K) treatments have demonstrated clinical benefits in other solid tumours and are key potential targets for clinical development against mGAC given the presence of recurrent alterations in these pathways. Furthermore, combination RTK/PI3K treatments may overcome compensatory mechanisms that arise using monotherapies, leading to improved patient outcomes. Herein, we investigated RTK/PI3K single and combination drug responses against our unique human mGAC-derived *PIK3CA* gain-of-function mutant, *human epidermal growth factor receptor 2 (HER2)*-negative, EGFR-expressing circulating tumour cell line, UWG02CTC, under two- and three-dimensional culture conditions to model different stages of metastasis. UWG02CTCs were highly responsive to the PI3K p110α-subunit targeted drugs PIK-75 (IC_50_ = 37.0 ± 11.1 nM) or alpelisib (7.05 ± 3.7 µM). Drug sensitivities were significantly increased in 3D conditions. Compensatory MAPK/ERK pathway upregulation by PI3K/Akt suppression was overcome by combination treatment with the EGFR inhibitor gefitinib, which was strongly synergistic. PIK-75 plus gefitinib significantly impaired UWG02CTC invasion in an organotypic assay. In conclusion, UWG02CTCs are a powerful ex vivo mGAC drug responsiveness model revealing EGFR/PI3K-targeted drugs as a promising combination treatment option for *HER2*-negative, *RAS* wild-type mGAC patients.

## 1. Introduction

Gastric cancer is the fifth most common cancer, accounting for the fourth highest rate of cancer-related mortality worldwide [1]. Gastric adenocarcinoma is the most common histological subtype of gastric cancer with an estimated 5-year survival rate of less than 30% [2]. Patients with advanced disease receive a combination of chemotherapy, immunotherapy and/or targeted treatments [3]. Despite advancements, the prognosis for advanced metastatic gastric adenocarcinoma (mGAC) remains poor due to the almost universal resistance to chemotherapy [4], and immunotherapy significantly benefits only a minority of patients [5]. Dysregulation of signalling pathways responsible for cell survival and cell death represents one of the main causes of chemoresistance [4]. New targeted treatment and/or combination treatments that block compensatory mechanisms contributing to drug resistance are needed.

**Figure 1:**
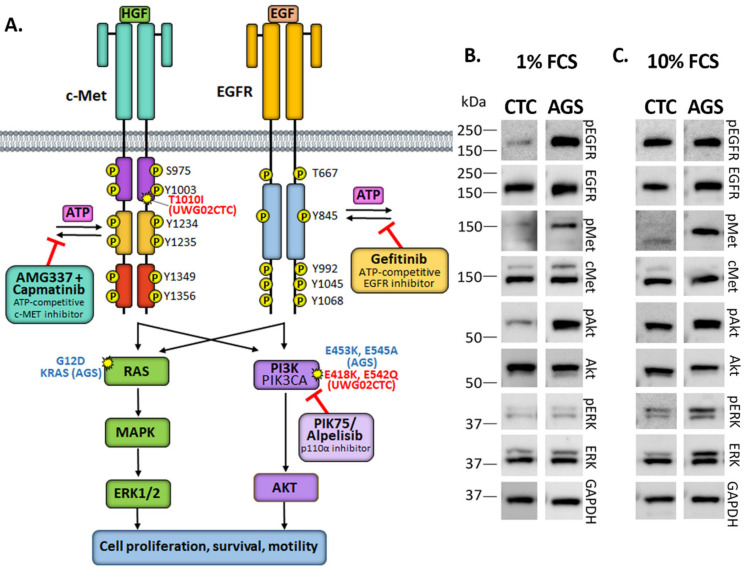
(**A**) Schematic diagram depicting the mesenchymal–epithelial transition factor (c-Met) and epidermal growth factor receptor (EGFR) signalling pathways with downstream mitogen-activated protein kinase (MAPK)/extracellular signal-regulated kinase (ERK) and phosphatidylinositol-3-kinase (PI3K)/Protein kinase B (Akt) signalling cascades. Indicated are the binding sites of all small molecule inhibitors used in this study and key mutations present in UWG02CTC and AGS cells. (**B**,**C**) Representative Western blots showing total and phosphorylated protein levels in (**B**) UWG02CTC and (**C**) AGS cells following overnight serum starvation (1% FCS) compared to cells grown in complete growth media (contains epidermal growth factor (EGF) and 10% foetal calf serum (FCS)) under 2D culture conditions.

Receptor Tyrosine Kinases (RTKs) and their downstream signalling pathways are often hyper-activated in malignancy and are the focus of new targeted treatment options [6,7,8]. These include the epidermal growth factor receptors (EGFRs) and human epidermal growth factor receptor 2 (HER2/ERBB2), the mesenchymal–epithelial transition factor (c-Met) and proteins in the phosphatidylinositol 3-kinases (PI3K)/Akt signalling pathway (Figure 1A). In gastric malignancies, *MET* mutations and/or gene amplifications occur in <5% of cases [9,10,11], while *EGFR* amplifications occur in 5–10% [12,13] and *HER2* amplifications occur in 20–30% [14,15,16,17]. These alterations trigger a range of downstream signalling pathways resulting in enhanced cancer cell proliferation, survival and migration/invasion (Figure 1A) [18,19,20]. Furthermore, an estimated 4–25% of GAC patients possess somatic mutations in *PIK3CA* [21], the gene encoding the p110α-subunit of PI3K, which leads to dysregulated PI3K/Akt activity [22,23]. Overexpression of *PIK3CA* and consequent Akt activation is also common among gastric malignancies [21,24,25]; thus, constituents of the PI3K/Akt pathway may serve as therapeutic targets for the treatment of gastric cancer. Several PI3K/Akt inhibitors are currently in routine clinical use for the treatment of cancers other than gastric cancer with alpelisib (also known as BYL719), a p110α-specific, small molecule PI3K inhibitor (Figure 1A), currently used in combination with hormone therapy in metastatic HER2-negative, *PIK3CA*-mutated breast cancer [26,27]. PIK-75 is a small molecule investigational drug that also selectively but more potently targets p110α (Figure 1A) [28]. No PI3K inhibitors are currently clinically approved for gastric cancer despite in vitro data suggesting efficacy in cell line models [29]. Other treatment strategies for gastric cancer may include targeting the cancer stem cell marker Lgr5. However, comprehensive strategies integrating multiple therapeutic modalities may be necessary to maximize efficacy and overcome potential resistance mechanisms [30].

Phase II clinical trials evaluating monotherapies directed at other targets such as EGFR have shown limited efficacy in gastric cancer [31], with no current treatments targeted at RTKs approved in HER2-negative gastric cancer. Gefitinib, a reversible, ATP-competitive tyrosine kinase inhibitor of EGFR (Figure 1A) in clinical use for non-small cell lung cancer [32,33] showed no benefit in a phase II clinical trial for patients with unselected gastroesophageal cancer [31] and has not been further pursued in this disease to date.

As upstream EGFR and c-Met ligand binding activates both the PI3K/Akt and the MAPK/ERK (extracellular-regulated kinase) pathways, crosstalk between these signalling pathways can occur, playing a critical role in drug resistance [34,35,36]. This paradigm has been successfully exploited in V600E mutant melanoma, where dual blockade with both BRAF and MEK inhibitors has led to significantly improved outcomes compared to monotherapy [37]. Thus, combination targeted therapies could be a valuable approach for mGAC, but better in vitro models of this disease are required. 

Circulating tumour cells (CTCs) are an extremely rare intermediate species of cells, which are shed from the primary tumour or existing metastases, enter the blood stream and form new metastases at distal sites [38]. To date, there are only about a dozen CTC lines established worldwide [39]. There is an increasing recognition of the limitations of using primary tumour features to guide systemic cancer treatment due to tumour heterogeneity and the frequent disparity observed between primary and metastatic sites. Thus, ex vivo expansion of CTCs provides a useful tool to study metastasis and responses to novel treatment strategies as they have unique intermediate characteristics between primary tumours and metastases. In a previous study, we comprehensively reported on the derivation and characterisation of a highly aggressive mGAC CTC line, UWG02CTC, which displayed a mixed adherent/loose aggregate mucinous phenotype under two-dimensional (2D) standard culture conditions [22]. This cell line harbours known gain-of-function hotspot mutations in *PIK3CA* (E418K and E542Q) and a mutation designated as having ‘conflicting classifications of pathogenicity’ (according to https://www.ncbi.nlm.nih.gov/clinvar/, accessed on 19 October 2023) in *MET* (T1010I) (in exon 14 c.3029C>T, p.Thr1010Ile, also identified as T992I [40]) (Figure 1A, Supplementary Table S1). While being HER2-negative, the UWG02CTC cell line expresses EGFR at both RNA and protein levels. In this study, we investigate the impact of these genetic alterations on the sensitivity and resistance to targeted PI3K, EGFR or c-Met drugs as either mono- or combination therapies using UWG02CTC as an ex vivo mGAC model. Using various culture conditions, we mimic different stages of metastasis: that is, 2D loose adherent (i.e., shedding primary tumour), 3D non-adherent (i.e., CTC in circulation) and cells embedded in collagen or hydrogel (i.e., primary or metastatic site) as well as organotypic invasion assays (i.e., invasion into new metastatic sites). 

## 2. Results

### 2.1. Monotherapy Effects

The gain-of-function *PIK3CA* mutation in UWG02CTC is suitable for the targeted small molecule inhibitors shown in Figure 1A. We therefore investigated the molecular and functional cellular consequences of these inhibitors against UWG02CTCs and the gastric adenocarcinoma cell line AGS, which also harbours *PIK3CA* and a *KRAS* mutation (Figure 1A, Supplementary Table S1), as a non-CTC but an mGAC cell line comparator. We first confirmed that both cell lines expressed the RTKs c-Met and EGFR, and downstream signalling molecules ERK and Akt under serum-starved (Figure 1B, left panel) or complete growth media (Figure 1B, right panel) two-dimensional (2D) conditions. In serum-starved (baseline) conditions, phospho-Met (pMet) and phospho-ERK (pERK) were present in both cell lines but at low levels (Figure 1B). Phospho-ERK levels were increased after culture of both cell lines in complete growth medium. Phospho-Akt (pAkt) was also evident at baseline, reflecting the presence of activating mutations in *PIK3CA* in both cell lines, but was enhanced in UWG02CTCs by complete growth media conditions (Figure 1B; Supplementary Figure S1). Phospho-EGFR levels were particularly high in AGS cells at baseline and were strongly enhanced in UWG02CTCs by culture in complete growth media (Figure 1B, Supplementary Figure S1). 

**Figure 2:**
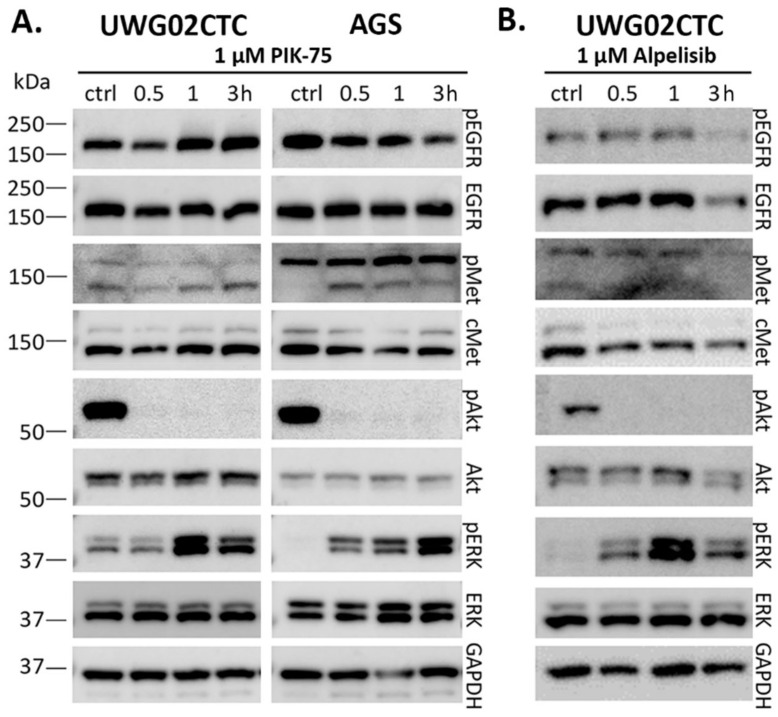
Effect of PI3K inhibitors on signalling responses in UWG02CTC and AGS cells. Representative Western blot images showing total and phosphorylated protein levels in response to treatment with (**A**) 1 µM PIK-75 on UWG02CTC and AGS cells over times indicated, and (**B**) 1 µM alpelisib on UWG02CTC cells over times indicated. Experiments were performed under complete growth media 2D culture conditions. The housekeeping protein GAPDH was used as a total protein loading control.

We then assessed the effect of the potent small molecule PI3K inhibitor PIK-75, and validated results with the clinically relevant small molecule PI3K inhibitor alpelisib on UWG02CTCs. Both cell lines were highly sensitive to PIK-75 with similar IC_50_ values (37.0–42.2 nM) under 2D culture conditions (Table 1). They also showed a significant reduction in pAkt levels compared to baseline (Figure 2A and Supplementary Figure S2). While PIK-75 had no significant effect on pEGFR and pMet, pERK was significantly upregulated upon PIK-75 treatment in both cell lines, confirming the compensatory effects of PI3K/Akt suppression via the MAPK/ERK pathway [41,42]. Alpelisib, though not as potent a cytotoxin as PIK-75 [43] (Table 1), returned IC_50_ values well within published ranges for stomach adenocarcinomas or oesophageal carcinomas [44] (range 1.4–225 µM; including data for AGS IC_50_ = 2.4 µM; www.cancerrxgene.org, accessed on 22 February 2024) and had similar effects on UWG02CTC signalling pathways (Figure 2B). The sensitivity of UWG02CTCs to PI3K/Akt pathway inhibition was confirmed using other cell viability and proliferation assays (Supplementary Figure S3A). 

**Figure 3:**
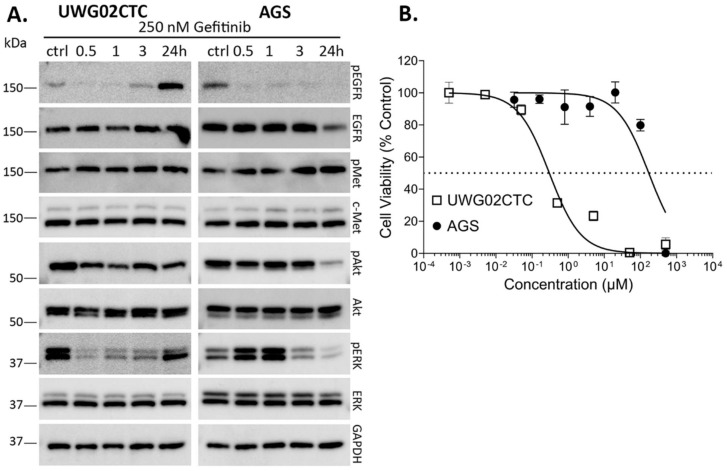
(**A**) Representative Western blot images of gefitinib-treated UWG02CTC and AGS cells under complete growth media 2D culture conditions (+EGF for UWG02CTCs). (**B**) Representative gefitinib dose–response curves of UWG02CTC (□) and AGS cells (●) (mean ± SEM, n = 3). Dotted line represents 50% cell viability. See Table 1 for IC_50_ values derived from multiple experiments.

Given the high levels of EGFR protein in both cell lines, we assessed the effect of the EGFR inhibitor gefitinib. AGS cells have a gain-of-function mutation in *KRAS*, a downstream effector in the EGFR pathway, leading to hyper-activation of ERK in these cells via resistance to upstream inhibition of EGFR [45], which was confirmed in our hands (Figure 3A). In contrast, treatment with gefitinib led to a rapid but transient reduction in EGFR and MAPK activation in UWG02CTCs with minimal effect on pMet or pAkt in both cell lines (Figure 3A, Supplementary Figure S4). AGS cells were >90-fold less sensitive to gefitinib due to their *KRAS* activating mutation.

The *MET* T1010I mutation in UWG02CTCs (Figure 1A) led us to examine any possible effects of c-Met kinase inhibitors capmatinib and AMG337 [46,47,48] (Figure 1A). This was compared to *MET* wild-type AGS and the *MET*-amplified SNU-5 mGAC cell lines. *MET* germline mutations have been linked to increased tumorigenicity in other cancers including colorectal [40]. Neither capmatinib nor AMG337 were cytotoxic (Supplementary Figures S5B,D and S6B,D) or cytostatic (Supplementary Figures S5C,E and S6C,E) against either cell line, likely due to the lack of *MET* amplification, which was confirmed via digital droplet PCR in both cell lines and RNA sequencing in UWG02CTC (Supplementary Figure S7A,B). By western blotting, minimal transient effects of capmatinib or AMG337 on the c-Met and PI3K/Akt signalling pathways but short-term stimulation of the MAPK pathway in both cell lines were observed (Supplementary Figures S5A and S6A). The potency of both drugs was confirmed with the SNU-5 mGAC cell line (IC_50_~1–2 nM; Supplementary Figure S7C,D) with AMG337 strongly reducing pMet levels (Supplementary Figure S7E). 

### 2.2. Effect of Drugs on UWG02CTC Invasion in 3D Organotypic Culture 

Other major roles of the PI3K/Akt, MAPK/ERK and c-Met signalling pathways are to promote cell migration and invasion [49,50,51]. The capacity of PIK-75, gefitinib and c-Met inhibition to impede UWG02CTC invasion was explored in an organotypic cell invasion assay, utilising fibroblast-contracted collagen matrices to recapitulate the tumour-stroma microenvironment of invasive tumour cells [52]. This assay is an excellent ex vivo tool for studies of cancer invasion and metastasis in a physiologically relevant microenvironment. 

**Figure 4:**
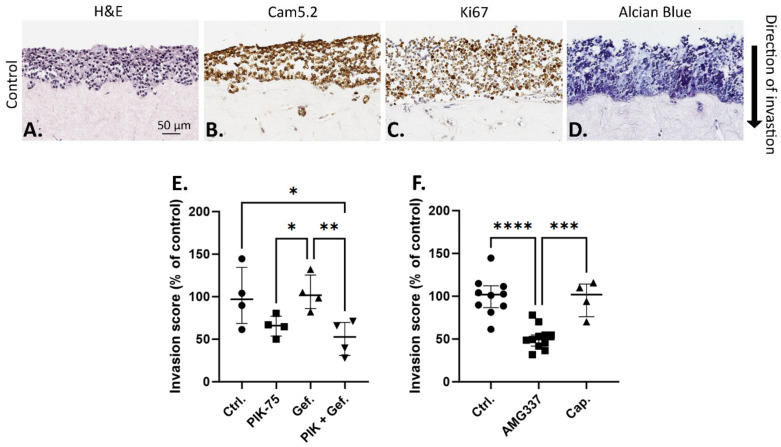
UWG02CTCs are invasive in 3D organotypic cultures. (**A**) Representative photo-micrographs of organotypic cultures showing direction of invasion over a 10–14-day time period through fibroblast-contracted collagen I matrices stained for (**A**) hematoxylin and eosin (H&E), (**B**) cytokeratin (Cam5.2), (**C**) Ki67 or (**D**) alcian blue (mucin) from untreated (no drugs added) matrices. Scale bare = 50 µm, all 4 images were taken at 20× magnification. (**E**,**F**) Invasion score calculated as average UWG02CTC cell count per matrix normalised to UWG02CTC invasion scores as percentage of control matrices. Drug concentrations used: 20 nM PIK-75, 125 nM gefitinib, 1 µM AMG337 and 1 µM capmatinib. Statistical analyses were performed using one-way ANOVA followed by a Fisher’s post hoc test. Values shown are median ± IQR; for (**E**), one experiment performed in quadruplicate, and for (**F**), three separate experiments performed at least in duplicate, with significance levels shown as *, *p* < 0.05; **, *p* ≤ 0.01; ***, *p* ≤ 0.001; ****, *p* ≤ 0.0001.

To the best of our knowledge, this is the first description of any such organotypic assay conducted using a CTC line, which together better recapitulates key aspects underlying tumour cell invasion and as a platform for identifying potential strategies for inhibiting metastasis. Under control complete growth media conditions (10% FCS plus EGF), UWG02CTC cells formed a multilayered sheet on top of the matrix with clear zones of invasion into the underlying matrix (Figure 4A). panCytokeratin staining verified the presence of invaded UWG02CTC cells in the matrix (Figure 4B) and that the cells forming the multilayered epithelial sheet on top of the matrix were proliferative as seen by uniform Ki67 staining (Figure 4C). In this model, the UWG02CTCs continued to secrete mucins (a hallmark of biologically functional gastric adenocarcinoma cells) (Figure 4D).

PIK-75 at half-IC_50_ concentration (chosen to limit cell death) showed a strong trend to reduce the invasive capacity of UWG02CTC compared to no drug control (Figure 4E). Gefitinib had no significant effect but in combination with PIK-75 significantly impaired invasive capacity (Figure 4E). Interestingly, while the presence of a high concentration of AMG337 caused a significant reduction in the invasion index compared to control matrices, capmatinib had no effect (Figure 4F). The reason for this is unclear but likely reflects differences in the pharmacological properties and target selectivity of AMG337 versus capmatinib, and the micro-environmental cues and signaling interactions present within the 3D tissue-like setting which may differentially influence the invasive capacity of cancer cells to c-Met inhibition.

### 2.3. Drug Responsiveness in 2D versus 3D Culture Models 

**Figure 5:**
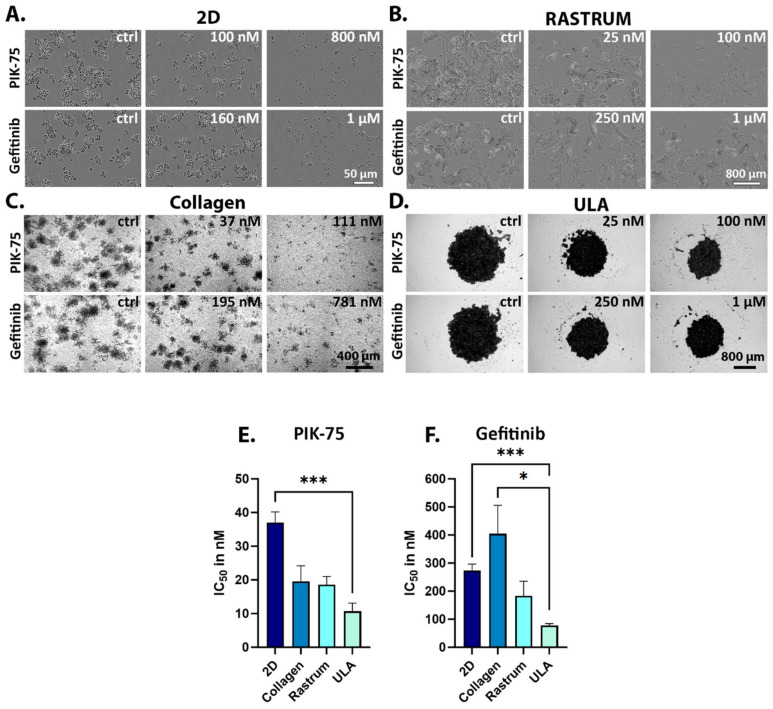
Drug responses of 2D- versus 3D-cultured UWG02CTCs. (**A**–**D**) Representative IncuCyte images of the different treatment conditions 72 h post drug addition on UWG02CTC cell morphology. (**E**,**F**) IC_50_ values derived from dose–response curves 72 h post drug addition. Values shown are mean ± SEM for PIK-75 n = 12 (2D), 2 (collagen), 3 (Rastrum) and 2 (ULA). Gefitinib n = 6 (2D), 4 (collagen), 2 (Rastrum) and 2 (ULA). Statistical significance was determined via Brown Forsythe ANOVA with Welch correction with significance levels defined as *, *p* < 0.05; *** *p* ≤ 0.001.

It is well appreciated that different cell culture conditions evoke different cellular responses [53]. UWG02CTC and AGS cell responsiveness to PIK-75, alpelisib, gefitinib, AMG337 and capmatinib was therefore investigated under various 2D and 3D cell culture conditions to model diverse tumour microenvironments. This included culturing in ULA plates, an environment that does not allow cells to attach to surfaces, thus promoting the loose aggregation of cells into clusters to simulate tumour cells in circulation. Levels of total and phosphorylated proteins in UW02CTCs grown under these conditions were generally similar to those seen in 2D culture conditions (Supplementary Figure S8). To mimic other steps of the metastatic cascade (i.e., invasion and metastasis formation), a collagen-based matrix and sophisticated 3D hydrogel bioprinting technology using the RASTRUM^TM^ 3D bioprinter (Inventia Life Science, Beaconsfield, Australia) was utilised. In the latter scenario, the UWG02CTCs were printed into a hydrogel matrix enriched with peptides to match the ECM stiffness (approx. 1.1 kPa) most closely resembling that of gastric and liver tissue, a common metastatic site for gastric cancer [54]. AGS cells did not grow well in these 3D matrices; comparisons were thus limited to 2D and ULA culture conditions (see Supplementary Figure S8A,B for all dose–response curves). Figure 5A–D depict representative images of the effect of different culturing conditions with increasing drug concentrations on UWG02CTC morphology and spheroid/cluster formation. In ULA plates, UWG02CTCs assemble into loosely aggregated spheroids (Figure 5D). UWG02CTCs adopt a spheroid structure with a ‘needle-cushion’ appearance when seeded into collagen matrices (Figure 5C) and grow well as loose clusters and aggregates when bioprinted into RASTRUM hydrogel matrices (Figure 5B). 

UWG02CTCs were more sensitive to PIK-75 in all 3D compared to 2D culture conditions (Figure 5E, Table 1), with no significant difference noted between these 3D conditions. There was a trend for increased sensitivity of UWG02CTCs to alpelisib in ULA versus 2D conditions though IC_50_ values were notably higher than those obtained for PIK-75 as expected (Table 1). AGS cell PIK-75 and gefitinib sensitivity was similar in ULA versus 2D culture conditions (Table 1). The UWG02CTCs were generally more sensitive to gefitinib in ULA versus the other conditions (Figure 5F). AMG337 and capmatinib were also ineffective against cell viability in 3D environments in both cell lines (Supplementary Figure S8). 

### 2.4. Combination Drug Analyses 

**Figure 6:**
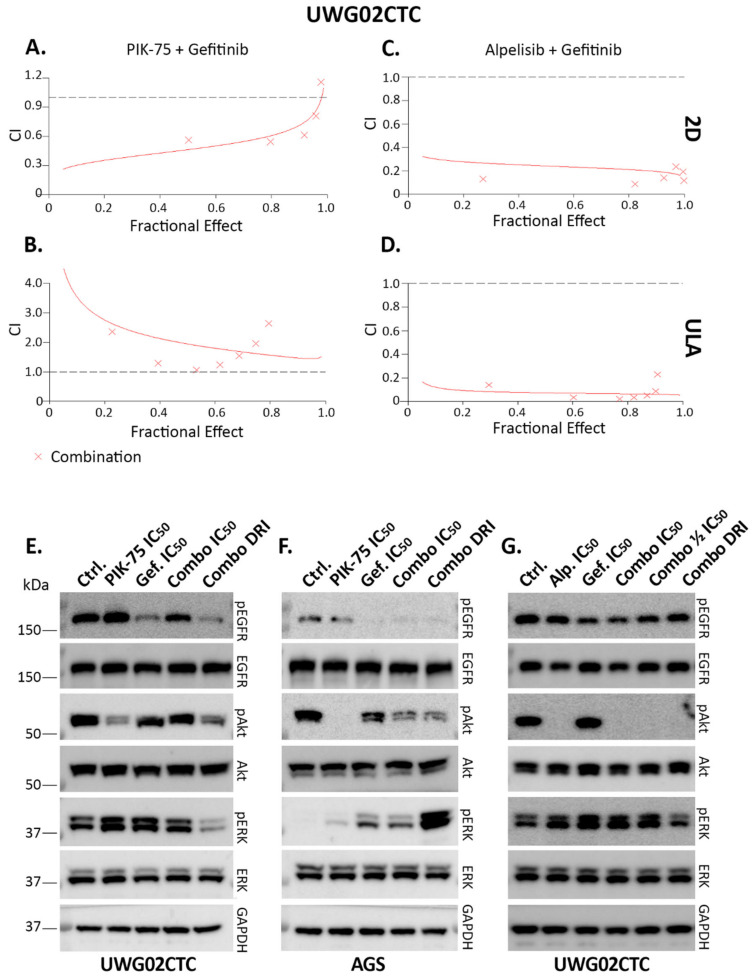
Combination drug treatment with PIK-75/alpelisib and gefitinib on UWG02CTCs. Fa-CI plots simulated by the median-effect equation for two drugs shown using CalcuSyn 2.11 software. (**A**,**B**) UWG02CTC with PIK-75+gefitinib in (**A**) 2D and (**B**) ULA conditions. (**C**,**D**) Treatment with alpesib and gefitinib in (**C**) 2D and (**D**) ULA conditions. Representative of at least 2 separate experiments each performed in triplicate. CI ≤ 1 synergistic, CI = 1 additive, CI ≥ 1 antagonistic. Dotted lines indicate CI = 1. (**E**–**G**) Western blot analysis of combination treatment on signalling pathway responses to either PIK-75 and gefitinib in (**E**) UWG02CTC or (**F**) AGS cells, and (**G**) alpelisib and gefitinib in UWG02CTC in 2D culture conditions. Cells were treated for 1 h with PIK-75 and gefitinib and for 3 h with alpelisib and gefitinib drug combinations at approximately either their IC_50_ concentrations derived from dose–response curves as single agents or their (experimental or calculated) DRI concentrations for Fa = 0.5 derived from combination drug experiments under 2D conditions. **E**) 40 nM PIK-75 + 250 nM gefitinib (IC_50_) or 16 nM PIK-75 + 20 nM gefitinib (DRI) n = 3. (**F**) 40 nM PIK-75 + 250 µM gefitinib (IC_50_) or 16 nM PIK-75 + 20 µM gefitinib (DRI) n = 3. (**G**) 6.6 µM alpelisib + 270 nM gefitinib (IC_50_), 3.3 µM alpelisib + 135 nM ½ IC_50_) or 600 nM alpelisib + 41 nM gefitinib (DRI).

To overcome the compensatory upregulation of pERK upon PIK-75 treatment, we trialled this drug in combination with gefitinib under 2D versus 3D conditions in UWG02CTCs. The Fractional Effect–Combination Index (Fa-CI) effect plots indicate a trend to synergism and meaningful dose reduction indices (DRIs) under 2D (Figure 6A) and RASTRUM culturing conditions (Supplementary Table S2A,C) (with most CI values < 1 at Fa values between 0.2–0.8). However, there was no synergistic effect for the combination treatment under ULA conditions (CI values either > 1 or = 1) (Figure 6B) and no benefit with respect to dose reduction of either drug (Supplementary Table S2B). This underscores the very high sensitivity of the cells to gefitinib and particularly PIK-75 as single agents under ULA conditions. In contrast, synergism was strong at all Fa values (average CI  < 0.3) with the alpelisib and gefitinib combination treatment in both 2D and ULA conditions (Figure 6C,D and Supplementary Table S2D,E). 

We then used the IC_50_ and DRI concentrations at Fa 0.5 for PIK-75 or the alpelisib and gefitinib combination calculated for UWG02CTCs in 2D conditions to analyse the effects on key signalling pathway proteins under these conditions via Western blotting (Figure 6E,G; Supplementary Figure S9 densitometry). Interestingly, pERK levels were reduced by DRI concentrations in UWG02CTCs but less so by the (higher) IC_50_ concentrations of PIK-75 and gefitinib either alone or in combination compared to controls. This indicates that gefitinib compensated for MAPK/ERK pathway upregulation by PI3K/Akt suppression, potentially leading to the synergistic effects on cell viability. Phospho-Akt levels were reduced with the PIK-75/gefitinib combination or PIK-75 (at IC_50_ concentration) alone compared to controls, and these levels were also reduced by any alpelisib/gefitinib combination or alpelisib (at IC_50_ concentration) alone (Figure 6E,G). Phospho-EGFR levels were strongly reduced by the DRI but not the (higher) IC_50_ concentrations of the PIK-75/gefitinib combination compared to controls, but this was not as apparent with the alpelisib/gefitinib combinations (Figure 6E,G). While pEGFR and pAkt levels were significantly reduced in AGS cells using the DRI PIK-75/gefitinib drug combination, the DRI concentration appeared to enhance pERK levels (Figure 6F). The reason for this is unclear since these drugs in combination were synergistic in terms of cell viability as indicated above.

Taken together, our results suggest that the combination of inhibiting both the EGFR and PI3K pathways could be a new treatment approach for mGAC patients harbouring *PIK3CA* mutations. 

## 3. Discussion

There is an urgent need for novel targeted and combination therapies in mGAC with existing approved treatments limited to the minority of patients who have HER2-positive disease. Targeted cancer treatments promise improved outcomes and reduced toxicities. However, the only clinically used targeted therapies for mGAC are directed against HER2 [15]. In this study we investigated new targeted treatment options for HER2-negative, *PI3KCA*-mutated mGAC using the circulating tumour cell line UWG02CTC and the HER2-negative, *PI3KCA*-mutated adenocarcinoma cell line AGS as a non-CTC but mGAC comparator cell line. 

Both cell lines were sensitive to PI3K inhibition using the highly selective and potent p100α PI3K subunit inhibitor experimental drug PIK-75 [55]. Although PIK-75 is not in clinical use due to its high toxicity profile, it has recently been revisited as a potential treatment option in T-cell acute lymphoblastic leukaemia [56]. We validated our findings in UWG02CTCs using the clinically approved PI3K inhibitor alpelisib, currently approved in combination with hormone therapy in advanced, HER2-negative, *PIK3CA*-mutated breast cancer [26]. Alpelisib is also under investigation in other solid malignancies [27], including in combination with chemotherapy in *PIK3CA*-mutated mGAC (ClinicalTrials.gov Identifier: NCT04526470). The high EGFR expression, HER2-negative and *KRAS* wild-type status of UWG02CTC also imparted sensitivity to gefitinib. However, it is important to note multiple phase III randomised control trials have failed to demonstrate a benefit for EGFR targeted monotherapy in unselected mGAC [57], and a more rational approach is required. A deeper understanding of the molecular pathway and likely resistance mechanisms (such as *RAS* status), and use of combination targeted therapies is likely required for improved clinical outcomes. 

Given the underwhelming clinical results of targeted monotherapies, the trend in targeted cancer treatment is toward combination regimens, largely to address the compensatory signalling mechanisms that occur when blocking one specific RTK [58]. Indeed, we also confirmed transient but strong MAPK/ERK pathway stimulation following PI3K/Akt suppression, which may limit effects on cell proliferation/survival especially if using the clinically available but less potent PI3K inhibitor alpelisib compared to PIK-75. Recent preclinical and clinical studies have demonstrated the potential benefits of combining PI3K inhibitors with EGFR inhibitors in other solid tumours, exhibiting synergistic effects by targeting multiple nodes within the interconnected EGFR/MAPK and PI3K/Akt signalling cascades, disrupting tumour growth and overcoming resistance mechanisms [59,60,61,62]. As a result, there are a number of active recruiting phase I/II clinical trials evaluating EGFR and PI3K inhibitors in other malignancies (ClinicalTrials.gov Identifier: NCT04495621, NCT05683418 NCT01816984) [61]. However, given a lack of data evaluating the potential efficacy of combined EGFR and PI3K inhibitor treatments for mGAC, comprehensive preclinical assessment is required, starting with drug response evaluation in both 2D and 3D cell culture conditions.

The development of 3D culture systems has become increasingly popular, as they more accurately capture the complexity of the tumour microenvironment [63,64]. Using our CTC line, we were able to mimic different stages of metastasis by culturing under either 2D loose adherent (i.e., shedding primary tumour), ULA (i.e., CTC in circulation), 3D via embedding in collagen or hydrogel (i.e., primary or metastatic site). We also utilised organotypic invasion assays to better mimic the tumour environment to study the effects of RTK and PI3K/Akt inhibition on the invasive capacity of the cells [63,65]. We found that UWG02CTCs, in contrast to AGS cells, are significantly more sensitive to PIK-75 or gefitinib as single agents in ULA conditions (i.e., under conditions mimicking CTCs in circulation) as compared to 2D culture conditions, suggestive of some innate feature of UWG02CTCs which predisposes them to PIK-75 or gefitinib when under conditions mimicking CTCs in circulation. This differential response has important therapeutic considerations. The highly potent effect seen in ULA conditions suggests monotherapy only is required to clear CTCs in circulation, while the synergistic effect seen in 2D culture conditions with PIK-75 and gefitinib may be required for activity against the primary tumour or established metastases. Targeting PI3K and EGFR pathways also significantly inhibited UWG02CTC invasion in organotypic assays, suggesting that PIK-75/gefitinib combination treatment would inhibit both cell viability/proliferation and hinder invasion into new metastatic sites [62,66,67,68]. Interestingly, alpelisib in combination with gefitinib was highly synergistic regardless of growth conditions, suggesting this particular combination would be necessary to best target the primary tumour or established metastases as well as CTCs in circulation. Finally, the observed drug response variations across diverse culturing conditions emphasize the necessity for careful consideration when interpreting preclinical drug studies for translation into animal models.

The c-Met pathway was of interest in this study as the UWG02CTCs harbour T1010I mutation [22] (also reported as *T992I*), which has been linked to decreased growth factor dependence and increased migration in various tumour cell lines [69,70]. That capmatinib or AMG337 did not impact cell proliferation in both UWG02CTCs and AGS was not surprising as both drugs are shown to be most effective (but not exclusively) in cells with *MET* amplification or exon 14 skipping mutation [46,71,72]. Furthermore, we found no synergistic or additive cytotoxic effects of capmatinib or AMG337 in combination with any other drugs used in this study. However, we did observe a significant effect of AMG337 on the invasive capacity of the UWG02CTCs in an organotypic invasion assay, which may be a result of interference with downstream effects of the T1010I mutation and/or inhibition of c-Met activation in general. The mutational allele frequency of the T1010I mutation in UWG02CTCs is ~50% [22], suggesting this mutation to be of germline origin. Unfortunately, we are unable to confirm this as no tissue or blood samples are available from the patient. Nonetheless, in a small cohort study of 22 metastatic gastric cancer patients, we detected T1010I *MET* germline mutations in 1/22 (4.5%) patients (Supplementary Figure S10), which is in line with other studies reporting germline T1010I mutations in 5.2% of colorectal cancer-affected sibling pairs and 4.1% in first-line relatives [40]. The role of this *MET* mutation in the pathogenesis of mGAC remains uncertain.

### Summary

In summary, our findings strongly indicate for the first time that the combination treatment with either PIK-75 or alpelisib with gefitinib is very effective against *PIK3CA*-mutated HER2-negative mGAC, a highly significant result given the paucity of targeted treatment options in HER2-negative mGAC. Furthermore, our study underscores the utility of UWG02CTC as a model for mGAC and the importance of utilising different culture conditions to predict drug responsiveness more accurately to better inform future animal preclinical testing.

## 4. Materials and Methods

### 4.1. Cell Culture

A patient-derived UWG02CTC gastric cancer circulating tumour cell line was maintained as described in Brungs et al., 2020 [22]. The AGS (CRL-1739) gastric cancer cell line (ATCC) was maintained at 37 °C in humidified 5% CO_2_ and 21% O_2_ normoxic conditions. Cells were grown in RPMI-1640 medium supplemented with 10% (*v*/*v*) FCS (Sigma-Aldrich, Macquarie Park, NSW, Australia), and 100 μg/mL penicillin/streptomycin (ThermoFisher Scientific, Loughborough, UK). All cells were subcultured at 80–90% confluence twice weekly, and routinely monitored for the absence of mycoplasma contamination. Short Tandem Repeat (STR) profiling was performed to reconfirm the identity of the cell lines [22].

### 4.2. Western Blotting

UWG02CTC and AGS cell lines seeded in either regular 2D culturing or ultra-low attachment surfaces were treated with AMG337, capmatinib, PIK-75, alpelisib or gefitinib (Focus Bioscience, St Lucia, QLD, Australia) at concentrations indicated in figure legends for 0.5, 1, 3 or 24 h, with vehicle control cells (0 h) treated with 0.1% DMSO. Cells were lysed in chilled RIPA buffer (50 mM Tris HCl, 150 mM NaCl, 1.0% TRITON-X100, 5 mM EDTA, 1 mM PMSF, 1 mM Na_3_VO_4_) and detached using a cell scraper. SDS-PAGE was performed under reducing conditions and PVDF membranes were probed with antibodies listed in Supplementary Table S3. Densitometry was performed using ImageJ version 1.53.

### 4.3. Cell Viability Assays

Cell viability assays were performed as previously described [22]. Briefly, UWG02CTC and AGS cells were seeded into 96-well microplates at densities of 15,000 or 10,000 cells per well, respectively. Cells were incubated with serial drug dilutions of AMG337, capmatinib, PIK-75, alpelisib or gefitinib, as single agents or in combination, for 72 h, with a final DMSO concentration of 0.1% across all wells. Cell viability was determined using the CellTitre 96 AQueous One Solution Cell Proliferation Assay (MTS) Kit (Promega, Alexandria, NSW, Australia) as per the manufacturer’s instructions. Light absorbance was measured at 490 nm using the SpectraMax Plus 384 Microplate Reader (Molecular Devices, Bio-Strategy, Campbellfield, VIC, Australia). Data were normalised against vehicle controls (0.1% DMSO) and plotted as a logarithmic sigmoidal dose–response curve with GraphPad Prism 9.0 from which IC_50_ (half maximal inhibitory concentration) values were interpolated.

Cell viability was also monitored using IncuCyte Cytotox Green reagent (Essen Bioscience, Sartorius, Dandenong South, VIC, Australia), a fluorescent dye, as per the manufacturer’s instructions. Images were acquired throughout 72 h drug incubations using the IncuCyte Zoom, 2016A, with images analysed using in-built software to evaluate the fluorescence of non-viable (green) cells at 524 nm. 

### 4.4. Organotypic Invasion Assays

Organotypic assays were performed, essentially as previously described [52], to investigate UWG02CTC invasion. Briefly, human skin-derived telomerase-immortalised fibroblasts (TIFs) [73] were resuspended in FCS at a concentration of 50,000 cells per matrix and combined with 2.5 mg/mL collagen type I, 10× minimal essential media (MEM) and 0.22 M NaOH. Following matrix contraction over seven days, 200,000 UWG02CTCs were seeded atop each matrix before transfer to an air–liquid interface. Matrices were exposed to treatment with 1 µM AMG337, 1 µM capmatinib, 125 nM gefitinib and 20 nM PIK-75, either as single agents or in combination, with control matrices treated with regular growth media. Following 10–14-day UWG02CTC invasion, matrices were processed, paraffin embedded and sectioned for histological analysis. Slides were stained with either haematoxylin and eosin (H&E) (POCD Scientific, North Rocks, NSW, Australia), pan cytokeratin (Cam5.2), Ki67 or alcian blue (performed at the Department of Pathology, Wollongong Hospital). Slides were imaged using an Aperio Digital Pathology Slide Scanner (Leica Biosystems, Hunter Medical Research Institute, New Lambton Heights, Australia) generating high-resolution images for the manual counting of UWG02CTC invasion into matrix tissue. UWG02CTC invasion was counted across the entire diameter and complete width of each matrix, with two to five matrices counted per control or drug treatment group for each experimental replicate. To obtain an invasion score, raw UWG02CTC cell counts across each matrix were normalised to a measure of UWG02CTC invasion per 1000 µm distance using the formula (raw invasion count/matrix length) × 1000 to correct for differences in matrix lengths. Data were further normalised by setting the control invasion score to 100, with all other treatment groups represented as relative percentage of the average control value. Significance of invasion was quantified using GraphPad Prism 9.0 to conduct a one-way ANOVA followed by a Fisher’s LSD post hoc test.

### 4.5. Collagen-Embedded Spheroids

An adaptation of organotypic culture [52] was used to promote UWG02CTC spheroid formation in a 3D in vitro environment. A collagen formulation comprised of 75.4% (*v*/*v*) 2.5 mg/mL collagen type I, 8.8% (*v*/*v*) 10× MEM and 8.8% (*v*/*v*) 0.22 M NaOH was combined with an 8.8% (*v*/*v*) FCS solution containing resuspended UWG02CTCs. Then, 100 µL containing the collagen–UWG02CTC mixture was aliquoted into a 96-well plate with approximately 2500 UWG02CTCs seeded per well. Following a 20 min incubation at 37 °C to allow collagen polymerisation to occur, 150 µL of UWG02CTC culture media was added to each well. After 24 h, serial drug dilutions were added to the collagen-embedded spheroids as described above for the cell viability assays. Drugs were added in sextuplicate, and cells were incubated for 5 days, with the refreshment of drug media after 3 days. Data were processed as described above for the 2D MTS assays.

### 4.6. RASTRUM Bioprinting

A RASTRUM 3D bioprinter (Inventia Life Science, Ingham Institute) was used for the printing of UWG02CTCs embedded in hydrogel matrices. Printing was performed as per the manufacturer’s instructions, using drop-on-demand bioprinting technology with bio-inks and activators. To start, the automated ‘greenlighting’ process for the priming of fluids into the printer nozzles and the sterilisation process were performed. UWG02CTCs were then passaged and resuspended in a specific activator at a density of 3.12 × 10^6^ cells/mL, before bio-inks and activators were aliquoted into a sterile cartridge for printing.

Matrix composition and stiffness was optimised, with a Px02.00 matrix with ~1.1 kPa stiffness containing no peptides or proteins used. Bioprinted 96-well plates were used for cell viability (MTS) assays as described above. 

## Figures and Tables

**Table 1:** IC_50_ of PIK-75, alpelisib and gefitinib or PIK-75, and gefitinib as single agents against UWG02CTC and AGS cells, respectively, under different culture conditions. Cells were incubated for 72 h (2D and ULA) or 96 h (collagen and RASTRUM) with drugs prior to analysis of cell viability.

Drug IC_50_ (Mean ± SEM)					
		Conditions			
Cell Line	Drug	2D	ULA	Collagen	Rastrum
UWG02CTC	PIK-75	37.0 ± 11.1 nM (n = 12)	10.7 ± 3.4 nM (n = 2) ^1^	19.5 ± 6.6 nM (n = 2)	18.6 ± 4.2 nM (n = 3)
	Alpelisib	7.05 ± 3.7 µM (n = 9)	3.7 ± 1.0 µM (n = 3)	ND	ND
	Gefitinib	273.0 ± 56.6 nM (n = 6)	77.2 ± 9.9 nM (n = 2) ^1^	404.6 ± 202.3 nM (n = 4) ^2^	182.8 ± 74.3 nM (n = 2)
AGS	PIK-75	42.4 ± 12.7 nM (n = 9)	30.4 ± 15.2 nM (n = 2)		
	Gefitinib	24.7 ± 15.6 µM (n = 4)	19.47 ± 5.4 µM (n = 4)		

ND = not determined. ^1^ *p* < 0.001 compared to 2D, ^2^ *p* < 0.01 compared to ULA.

## Data Availability

All data generated or analysed during this study are included in this published article and its supplementary information files.

## Supplementary Materials

The following supporting information can be downloaded at: https://www.mdpi.com/article/10.3390/ijms25105565/s1.

## References

[1] Sung H., Ferlay J., Siegel R.L., Laversanne M., Soerjomataram I., Jemal A., Bray F. (2021). Global cancer statistics 2020: GLOBOCAN estimates of incidence and mortality worldwide for 36 cancers in 185 countries. CA A Cancer J. Clin..

[2] Lin Y.M., Chiu N.C., Li A.F., Liu C.A., Chou Y.H., Chiou Y.Y. (2017). Unusual gastric tumors and tumor-like lesions: Radiological with pathological correlation and literature review. World J. Gastroenterol..

[3] Lordick F., Carneiro F., Cascinu S., Fleitas T., Haustermans K., Piessen G., Vogel A., Smyth E.C., on behalf of theESMO Guidelines Committee (2022). Gastric cancer: ESMO Clinical Practice Guideline for diagnosis, treatment and follow-up. Ann. Oncol..

[4] Shi W.J., Gao J.B. (2016). Molecular mechanisms of chemoresistance in gastric cancer. World J. Gastrointest. Oncol..

[5] Rha S.Y., Oh D., Yañez P., Bai Y.X., Ryu M.H., Lee J.Y., Rivera F., Alves G.V., Garrido M., Shiu K.K. (2023). Pembrolizumab plus chemotherapy versus placebo plus chemotherapy for HER2-negative advanced gastric cancer (KEYNOTE-859): A multicentre, randomised, double-blind, phase 3 trial. Lancet Oncol..

[6] Teng L.S., Lu J. (2013). cMET as a potential therapeutic target in gastric cancer. Int. J. Mol. Med..

[7] Takeuchi K., Ito F. (2011). Receptor tyrosine kinases and targeted cancer therapeutics. Biol. Pharm. Bull..

[8] Pottier C., Fresnais M., Gilon M., Jerusalem G., Longuespee R., Sounni N.E. (2020). Tyrosine Kinase Inhibitors in Cancer: Breakthrough and Challenges of Targeted Therapy. Cancers.

[9] El Darsa H., El Sayed R., Abdel-Rahman O. (2020). MET Inhibitors for the Treatment of Gastric Cancer: What’s Their Potential?. J. Exp. Pharmacol..

[10] Janjigian Y.Y., Tang L.H., Coit D.G., Kelsen D.P., Francone T.D., Weiser M.R., Jhanwar S.C., Shah M.A. (2011). MET expression and amplification in patients with localized gastric cancer. Cancer Epidemiol. Biomark. Prev..

[11] Kawakami H., Okamoto I. (2016). MET-targeted therapy for gastric cancer: The importance of a biomarker-based strategy. Gastric Cancer.

[12] Smyth E.C., Vlachogiannis G., Hedayat S., Harbery A., Hulkki-Wilson S., Salati M., Kouvelakis K., Fernandez-Mateos J., Cresswell G.D., Fontana E. (2021). EGFR amplification and outcome in a randomised phase III trial of chemotherapy alone or chemotherapy plus panitumumab for advanced gastro-oesophageal cancers. Gut.

[13] Nakamura Y., Sasaki A., Yukami H., Jogo T., Kawazoe A., Kuboki Y., Taniguchi H., Yamashita R., Kuwata T., Ozawa M. (2020). Emergence of concurrent multiple EGFR mutations and MET amplification in a patient with EGFR-amplified advanced gastric cancer treated with cetuximab. JCO Precis. Oncol..

[14] Kelly C.M., Janjigian Y.Y. (2016). The genomics and therapeutics of HER2-positive gastric cancer-from trastuzumab and beyond. J. Gastrointest. Oncol..

[15] Bang Y.J., Van Cutsem E., Feyereislova A., Chung H.C., Shen L., Sawaki A., Lordick F., Ohtsu A., Omuro Y., Satoh T. (2010). Trastuzumab in combination with chemotherapy versus chemotherapy alone for treatment of HER2-positive advanced gastric or gastro-oesophageal junction cancer (ToGA): A phase 3, open-label, randomised controlled trial. Lancet.

[16] Gravalos C., Jimeno A. (2008). HER2 in gastric cancer: A new prognostic factor and a novel therapeutic target. Ann. Oncol..

[17] Prins M.J., Ruurda J.P., van Diest P.J., van Hillegersberg R., Ten Kate F.J. (2013). The significance of the HER-2 status in esophageal adenocarcinoma for survival: An immunohistochemical and an in situ hybridization study. Ann. Oncol..

[18] Bradley C.A., Salto-Tellez M., Laurent-Puig P., Bardelli A., Rolfo C., Tabernero J., Khawaja H.A., Lawler M., Johnston P.G., Van Schaeybroeck S. (2017). Targeting c-MET in gastrointestinal tumours: Rationale, opportunities and challenges. Nat. Rev. Clin. Oncol..

[19] Keller S., Kneissl J., Grabher-Meier V., Heindl S., Hasenauer J., Maier D., Mattes J., Winter P., Luber B. (2017). Evaluation of epidermal growth factor receptor signaling effects in gastric cancer cell lines by detailed motility-focused phenotypic characterization linked with molecular analysis. BMC Cancer.

[20] Fruman D.A., Chiu H., Hopkins B.D., Bagrodia S., Cantley L.C., Abraham R.T. (2017). The PI3K Pathway in Human Disease. Cell.

[21] Matsuoka T., Yashiro M. (2018). Biomarkers of gastric cancer: Current topics and future perspective. World J. Gastroenterol..

[22] Brungs D., Minaei E., Piper A.K., Perry J., Splitt A., Carolan M., Ryan S., Wu X.J., Corde S., Tehei M. (2020). Establishment of novel long-term cultures from EpCAM positive and negative circulating tumour cells from patients with metastatic gastroesophageal cancer. Sci. Rep..

[23] Kim J.-W., Lee H.S., Nam K.H., Ahn S., Kim J.W., Ahn S.-H., Park D.J., Kim H.-H., Lee K.-W. (2017). PIK3CA mutations are associated with increased tumor aggressiveness and Akt activation in gastric cancer. Oncotarget.

[24] Ito C., Nishizuka S.S., Ishida K., Uesugi N., Sugai T., Tamura G., Koeda K., Sasaki A. (2017). Analysis of PIK3CA mutations and PI3K pathway proteins in advanced gastric cancer. J. Surg. Res..

[25] Ye D.M., Xu G., Ma W., Li Y., Luo W., Xiao Y., Liu Y., Zhang Z. (2020). Significant function and research progress of biomarkers in gastric cancer. Oncol. Lett..

[26] Andre F., Ciruelos E., Rubovszky G., Campone M., Loibl S., Rugo H.S., Iwata H., Conte P., Mayer I.A., Kaufman B. (2019). Alpelisib for PIK3CA-Mutated, Hormone Receptor-Positive Advanced Breast Cancer. N. Engl. J. Med..

[27] Juric D., Rodon J., Tabernero J., Janku F., Burris H.A., Schellens J.H.M., Middleton M.R., Berlin J., Schuler M., Gil-Martin M. (2018). Phosphatidylinositol 3-Kinase alpha-Selective Inhibition With Alpelisib (BYL719) in PIK3CA-Altered Solid Tumors: Results From the First-in-Human Study. J. Clin. Oncol..

[28] Ma X., Hu Y. (2013). Targeting PI3K/Akt/mTOR cascade: The medicinal potential, updated research highlights and challenges ahead. Curr. Med. Chem..

[29] Zhong Z., Wang T., Zang R., Zang Y., Feng Y., Yan S., Geng C., Zhu N., Wang Q. (2024). Dual PI3K/mTOR inhibitor PF-04979064 regulates tumor growth in gastric cancer and enhances drug sensitivity of gastric cancer cells to 5-FU. Biomed. Pharmacother..

[30] He J., Han J., Lin K., Wang J., Li G., Li X., Gao Y. (2023). PTEN/AKT and Wnt/beta-catenin signaling pathways regulate the proliferation of Lgr5+ cells in liver cancer. Biochem. Biophys. Res. Commun..

[31] Adelstein D.J., Rodriguez C.P., Rybicki L.A., Ives D.I., Rice T.W. (2012). A phase II trial of gefitinib for recurrent or metastatic cancer of the esophagus or gastroesophageal junction. Investig. New Drugs.

[32] Kazandjian D., Blumenthal G.M., Yuan W., He K., Keegan P., Pazdur R. (2016). FDA Approval of Gefitinib for the Treatment of Patients with Metastatic EGFR Mutation-Positive Non-Small Cell Lung Cancer. Clin. Cancer Res..

[33] Tamura K., Yoshida T., Masuda K., Matsumoto Y., Shinno Y., Okuma Y., Goto Y., Horinouchi H., Yamamoto N., Ohe Y. (2023). Comparison of clinical outcomes of osimertinib and first-generation EGFR-tyrosine kinase inhibitors (TKIs) in TKI-untreated EGFR-mutated non-small-cell lung cancer with leptomeningeal metastases. ESMO Open.

[34] Guo A., Villen J., Kornhauser J., Lee K.A., Stokes M.P., Rikova K., Possemato A., Nardone J., Innocenti G., Wetzel R. (2008). Signaling networks assembled by oncogenic EGFR and c-Met. Proc. Natl. Acad. Sci. USA.

[35] Liu X., Wang Q., Yang G., Marando C., Koblish H.K., Hall L.M., Fridman J.S., Behshad E., Wynn R., Li Y. (2011). A novel kinase inhibitor, INCB28060, blocks c-MET-dependent signaling, neoplastic activities, and cross-talk with EGFR and HER-3. Clin. Cancer Res..

[36] Puri N., Salgia R. (2008). Synergism of EGFR and c-Met pathways, cross-talk and inhibition, in non-small cell lung cancer. J. Carcinog..

[37] Robert C., Karaszewska B., Schachter J., Rutkowski P., Mackiewicz A., Stroiakovski D., Lichinitser M., Dummer R., Grange F., Mortier L. (2015). Improved Overall Survival in Melanoma with Combined Dabrafenib and Trametinib. N. Engl. J. Med..

[38] Pantel K., Alix-Panabieres C. (2010). Circulating tumour cells in cancer patients: Challenges and perspectives. Trends Mol. Med..

[39] Smit D.J., Pantel K., Jucker M. (2021). Circulating tumor cells as a promising target for individualized drug susceptibility tests in cancer therapy. Biochem. Pharmacol..

[40] Neklason D.W., Done M.W., Sargent N.R., Schwartz A.G., Anton-Culver H., Griffin C.A., Ahnen D.J., Schildkraut J.M., Tomlinson G.E., Strong L.C. (2011). Activating mutation in MET oncogene in familial colorectal cancer. Bmc Cancer.

[41] Chandarlapaty S., Sawai A., Scaltriti M., Rodrik-Outmezguine V., Grbovic-Huezo O., Serra V., Majumder P.K., Baselga J., Rosen N. (2011). AKT inhibition relieves feedback suppression of receptor tyrosine kinase expression and activity. Cancer Cell.

[42] Serra V., Scaltriti M., Prudkin L., Eichhorn P.J., Ibrahim Y.H., Chandarlapaty S., Markman B., Rodriguez O., Guzman M., Rodriguez S. (2011). PI3K inhibition results in enhanced HER signaling and acquired ERK dependency in HER2-overexpressing breast cancer. Oncogene.

[43] Chang D.Y., Ma W.L., Lu Y.S. (2021). Role of Alpelisib in the Treatment of PIK3CA-Mutated Breast Cancer: Patient Selection and Clinical Perspectives. Ther. Clin. Risk Manag..

[44] Kim K.J., Kim J.W., Sung J.H., Suh K.J., Lee J.Y., Kim S.H., Lee J.O., Kim J.W., Kim Y.J., Kim J.H. (2020). PI3K-targeting strategy using alpelisib to enhance the antitumor effect of paclitaxel in human gastric cancer. Sci. Rep..

[45] Tria S.M., Burge M.E., Whitehall V.L.J. (2023). The Therapeutic Landscape for KRAS-Mutated Colorectal Cancers. Cancers.

[46] Hughes P.E., Rex K., Caenepeel S., Yang Y., Zhang Y., Broome M.A., Kha H.T., Burgess T.L., Amore B., Kaplan-Lefko P.J. (2016). In Vitro and In Vivo Activity of AMG 337, a Potent and Selective MET Kinase Inhibitor, in MET-Dependent Cancer Models. Mol. Cancer Ther..

[47] Wang Y., Jin R. (2018). The research progress of c-Met inhibitors in clinical trials. Canc Cell Res..

[48] Du Z., Caenepeel S., Shen Y., Rex K., Zhang Y., He Y., Tang E.T., Wang O., Zhong W., Zhou H. (2016). Preclinical Evaluation of AMG 337, a Highly Selective Small Molecule MET Inhibitor, in Hepatocellular Carcinoma. Mol. Cancer Ther..

[49] Zhang Y., Xia M., Jin K., Wang S., Wei H., Fan C., Wu Y., Li X., Li X., Li G. (2018). Function of the c-Met receptor tyrosine kinase in carcinogenesis and associated therapeutic opportunities. Mol. Cancer.

[50] Yang J., Nie J., Ma X., Wei Y., Peng Y., Wei X. (2019). Targeting PI3K in cancer: Mechanisms and advances in clinical trials. Mol. Cancer.

[51] Hervieu A., Kermorgant S. (2018). The Role of PI3K in Met Driven Cancer: A Recap. Front. Mol. Biosci..

[52] Timpson P., McGhee E.J., Erami Z., Nobis M., Quinn J.A., Edward M., Anderson K.I. (2011). Organotypic collagen I assay: A malleable platform to assess cell behaviour in a 3-dimensional context. J. Vis. Exp..

[53] Chaicharoenaudomrung N., Kunhorm P., Noisa P. (2019). Three-dimensional cell culture systems as an in vitro platform for cancer and stem cell modeling. World J. Stem Cells.

[54] Cox T.R., Erler J.T. (2011). Remodeling and homeostasis of the extracellular matrix: Implications for fibrotic diseases and cancer. Dis. Model. Mech..

[55] Sa J.K., Hong J.Y., Lee I.K., Kim J.S., Sim M.H., Kim H.J., An J.Y., Sohn T.S., Lee J.H., Bae J.M. (2020). Comprehensive pharmacogenomic characterization of gastric cancer. Genome Med..

[56] Lim F.Q., Chan A.S., Yokomori R., Huang X.Z., Theardy M.S., Yeoh A.E.J., Tan S.H., Sanda T. (2023). Targeting dual oncogenic machineries driven by TAL1 and PI3K-AKT pathways in T-cell acute lymphoblastic leukemia. Haematologica.

[57] Maron S.B., Xu J., Janjigian Y.Y. (2020). Targeting EGFR in Esophagogastric Cancer. Front. Oncol..

[58] Sorokin A.V., Marie P.K., Bitner L., Syed M., Woods M., Manyam G., Kwong L.N., Johnson B., Morris V., Jones P. (2022). Targeting RAS Mutant Colorectal Cancer with Dual Inhibition of MEK and CDK4/6. Cancer Res..

[59] Chen D., Hong R., Cao Y., Wu Q., Wang Y., Chen J., Li J., Zhang W., Zhan Q. (2023). Combined Wee1 and EGFR inhibition reveals synergistic antitumor effect in esophageal squamous cell carcinoma. Carcinogenesis.

[60] Li G., Song Z., Ru Y., Zhang J., Luo L., Yang W., Wu H., Jin H., Bao X., Wei D. (2023). Small-molecule nanoprodrug with high drug loading and EGFR, PI3K/AKT dual-inhibiting properties for bladder cancer treatment. Exploration.

[61] Brisson R.J., Kochanny S., Arshad S., Dekker A., DeSouza J.A., Saloura V., Vokes E.E., Seiwert T.Y. (2019). A pilot study of the pan-class I PI3K inhibitor buparlisib in combination with cetuximab in patients with recurrent or metastatic head and neck cancer. Head. Neck.

[62] Yu Y., Xiao Z., Lei C., Ma C., Ding L., Tang Q., He Y., Chen Y., Chang X., Zhu Y. (2023). BYL719 reverses gefitinib-resistance induced by PI3K/AKT activation in non-small cell lung cancer cells. Bmc Cancer.

[63] Alzeeb G., Metges J.-P., Corcos L., Jossic-Corcos L. (2020). Three-dimensional culture systems in gastric cancer research. Cancers.

[64] Fontoura J.C., Viezzer C., Dos Santos F.G., Ligabue R.A., Weinlich R., Puga R.D., Antonow D., Severino P., Bonorino C. (2020). Comparison of 2D and 3D cell culture models for cell growth, gene expression and drug resistance. Mater. Sci. Eng. C Mater. Biol. Appl..

[65] Duval K., Grover H., Han L.-H., Mou Y., Pegoraro A.F., Fredberg J., Chen Z. (2017). Modeling physiological events in 2D vs. 3D cell culture. Physiology.

[66] Li J., Kleeff J., Giese N., Buchler M.W., Korc M., Friess H. (2004). Gefitinib (‘Iressa’, ZD1839), a selective epidermal growth factor receptor tyrosine kinase inhibitor, inhibits pancreatic cancer cell growth, invasion, and colony formation. Int. J. Oncol..

[67] Zhen Y., Guanghui L., Xiefu Z. (2014). Knockdown of EGFR inhibits growth and invasion of gastric cancer cells. Cancer Gene Ther..

[68] Yamaguchi H., Yoshida S., Muroi E., Yoshida N., Kawamura M., Kouchi Z., Nakamura Y., Sakai R., Fukami K. (2011). Phosphoinositide 3-kinase signaling pathway mediated by p110alpha regulates invadopodia formation. J. Cell Biol..

[69] Ma P.C., Kijima T., Maulik G., Fox E.A., Sattler M., Griffin J.D., Johnson B.E., Salgia R. (2003). c-MET mutational analysis in small cell lung cancer: Novel juxtamembrane domain mutations regulating cytoskeletal functions. Cancer Res..

[70] Liu S.Y., Meric-Bernstam F., Parinyanitikul N., Wang B.L., Eterovic A.K., Zheng X.F., Gagea M., Chavez-MacGregor M., Ueno N.T., Lei X.D. (2015). Functional consequence of the MET-T1010I polymorphism in breast cancer. Oncotarget.

[71] Wolf J., Seto T., Han J.Y., Reguart N., Garon E.B., Groen H.J.M., Tan D.S.W., Hida T., de Jonge M., Orlov S.V. (2020). Capmatinib in MET Exon 14-Mutated or MET-Amplified Non-Small-Cell Lung Cancer. N. Engl. J. Med..

[72] Choi W., Park S.Y., Lee Y., Lim K.Y., Park M., Lee G.K., Han J.Y. (2021). The Clinical Impact of Capmatinib in the Treatment of Advanced Non-Small Cell Lung Cancer with MET Exon 14 Skipping Mutation or Gene Amplification. Cancer Res. Treat..

[73] Harris N.L.E., Vennin C., Conway J.R.W., Vine K.L., Pinese M., Cowley M.J., Shearer R.F., Lucas M.C., Herrmann D., Allam A.H. (2017). SerpinB2 regulates stromal remodelling and local invasion in pancreatic cancer. Oncogene.

