# Supplementary material for: EGFR and PI3K Signalling Pathways as Promising Targets on Circulating Tumour Cells from Patients with Metastatic Gastric Adenocarcinoma
Source: Int J Mol Sci. 2024 May 20;25(10):5565. doi: 10.3390/ijms25105565 (PMC11122469; doi:10.3390/ijms25105565)
Supplement: Supplementary file 1 [file ijms-25-05565-s001.zip › ijms-2992576-supplementary.pdf]

Additional File 1

Supplementary Tables

Table S1: All reported mutations in UWG02CTC and AGS cell lines

Gene	Encoded protein	Mutation	
		**UWG02CTC**	**AGS**
CTNNB1	β-Catenin	Gain-of-function<br>S33F	reduced degradation<br>G34E (c.101G>A)
PIK3CA	PIK3 p110α catalytic subunit	Gain-of-function<br>E418K, E542K	Gain-of-function<br>E453K (c.1357G>A),<br>E545A (c.1634A>C)
MET	Hepatic growth factor receptor	Gain-of-function<br>T1010I	/
CREBBP	CREB binding protein	Loss-of-function<br>R1446C	/
SMAD4	SMAD family member 4	Loss-of-function<br>D537H	/
GNAS	Guanine nucleotide binding protein	Gain-of-function<br>R201H	/
CDH1	E-Cadherin	/	truncating loss of protein<br>G579fs*9<br>(c.1733_1734insC)
KRAS	GTPase KRas	/	Gain-of-function<br>G12D (c.35G>A)

Both cell lines are HER2-negative; for the patient from whom the UWG02CTC line was derived this was determined via *in situ* hybridisation.

Table S2: Drug combination experimental values (from representative experiments)

A.

UWG02CTC – 2D					
Concentration		Fa-CI		DRI	
**Gefitinib (nM)**	**PIK-75 (nM)**	**Fa**	**CI**	**Gefitinib**	**PIK-75**
125	25	0.50316	0.564	11.681	2.089
250	50	0.796305	0.545	11.466	2.184
500	100	0.918814	0.615	9.749	1.951
1000	200	0.960622	0.814	7.153	1.484
2000	400	0.97861	1.160	4.896	1.046

DRI = Dose reduction Index, CI = combination index, Fa = effect,
CI < 1 Synergistic, CI = 1 Additive, CI > 1 Antagonistic

B.

UWG02CTC – ULA				
Concentration		Fa-CI		DRI
**Gefitinib (nM)**	**PIK-75 (nM)**	**Fa**	**CI**	NA
15.625	0.390625	0.0747064	10.114	
31.25	0.78125	0.226788	2.333	
62.5	1.5625	0.397545	1.251	
125	3.125	0.53095	1.065	
250	6.25	0.623266	1.183	
250	6.25	0.623266	1.183	
500	12.5	0.6873	1.533	
1000	25	0.750267	1.915	

C.

UWG02CTC – Rastrum					
Concentration		Fa-CI		DRI	
**PIK-75 (nM)**	**Gefitinib (nM)**	**Fa**	**CI**	**PIK-75**	**Gefitinib**
0.488281	39.0625	0.157098	1.281	14.782	0.824
1.95313	156.25	0.606757	0.412	13.465	2.963
7.8125	625	0.789815	0.612	5.802	2.277
31.25	2500	0.986187	0.157	8.785	23.348
125	10000	0.997504	0.178	6.298	51.243

Table S2 continued:

D.

UWG02CTC – 2D					
Concentration		Fa-CI		DRI	
**Gefitinib (nM)**	**Alpelisib (nM)**	**Fa**	**CI**	**Gefitinib**	**Alpelisib (nM)**
205.761	205.761	0.271943	0.131	20.898	12.097
617.284	617.284	0.821499	0.086	30.384	18.826
1851.85	1851.85	0.927022	0.140	18.370	11.699
5555.56	5555.56	0.970907	0.234	10.790	7.054
16666.7	16666.7	0.996548	0.192	12.749	8.836
50000	50000	0.999753	0.117	20.016	14.901

E.

UWG02CTC – ULA					
Concentration		Fa-CI		DRI	
**Gefitinib (nM)**	**Alpelisib (nM)**	**Fa**	**CI**	**Gefitinib**	**Alpelisib**
68.5871	68.5871	0.294586	0.141	8.407	45.777
205.761	205.761	0.603243	0.033	31.705	547.268
617.284	617.284	0.770266	0.022	46.644	1631.251
1851.85	1851.85	0.822576	0.036	28.570	1334.459
5555.56	5555.56	0.870909	0.053	1257.457	19.259
16666.7	16666.7	0.901499	0.089	975.642	11.381

AGS

Table S3: Primary and secondary antibodies used for western blotting and immunohistochemistry.

Antibody Type	Target	Host species	Dilution used*	Supplier and catalogue number
**Primary**	EGF Receptor (D38B1)	Rabbit mAb	1:2000*	Cell Signaling Technology #4267
	Phospho-EGF Receptor (Tyr1068) (D7A5) XP	Rabbit mAb	1:1000*	Cell Signaling Technology #3777
	c-MET (D1C2) XP	Rabbit mAb	1:2000*	Cell Signaling Technology #8198
	Phospho-MET (Tyr1234/1235) (D26) XP	Rabbit mAb	1:1000*	Cell Signaling Technology #3077
	AKT (pan) (11E7)	Rabbit mAb	1:2000*	Cell Signaling Technology #4685
	Phospho-AKT (Ser473) (D9E) XP	Rabbit mAb	1:2000*	Cell Signaling Technology #4060
	p44/42 MAPK (Erk1/2) (137F5)	Rabbit mAb	1:2000*	Cell Signaling Technology #4695
	Phospho-p44/42 MAPK (Erk1/2) (Thr202/Tyr204) (D13.14.4E) XP	Rabbit mAb	1:2000*	Cell Signaling Technology #4370
	GAPDH	Mouse mAb	1:10,000*	Sigma #G8795
	B-Catenin (D10A8) XP	Rabbit mAb	1:2000*	Cell Signaling Technology #8480
	E-Cadherin (M168) C-terminal	Mouse mAb	1:2000*	Abcam #ab76055
	EpCAM (Ber-EP4)	Mouse mAb	1:2000*	Abcam #ab7504
	SLUG	Rabbit pAb	1:2000*	Abcam #ab27568
	N-Cadherin	Mouse mAb	1:2000*	Sigma #C3865
	Vimentin (EPR3776)	Rabit mAb	1:2000*	Abcam #ab92547
	ZEB1 (BLR102H)	Rabbit mAb	1:2000*	Abcam #ab276129
	CD44 (E7K2Y) XP	Rabbit mAb	1:2000*	Cell Signaling Technology #37259
	Focal Adhesion Kinase, clone EP695Y	Rabbit mAb	1:2000*	Millipore Sigma #04-591
	Phospho-Focal Adhesion Kinase (Tyr397), clone 18	Mouse mAb	1:2000*	Millipore Sigma #05-1140
	Ki67 (Dako Omnis)	Mouse mAb	1:40	Agilent #MIB-1
	CAM5.2 (cytokeratin)	Mouse mAb	1:100	Leica #NCL-L-5D3
**Secondary**	Goat Anti-Rabbit IgG, HRP-linked	Goat	1:5000*	Cell Signaling Technology #7074
	Goat Anti-Mouse IgG HRP-linked	Goat	1:5000*	Abcam #ab205719

* Diluted in 2% (w/v) skim milk powder in Tris-buffered saline solution

Supplementary figures

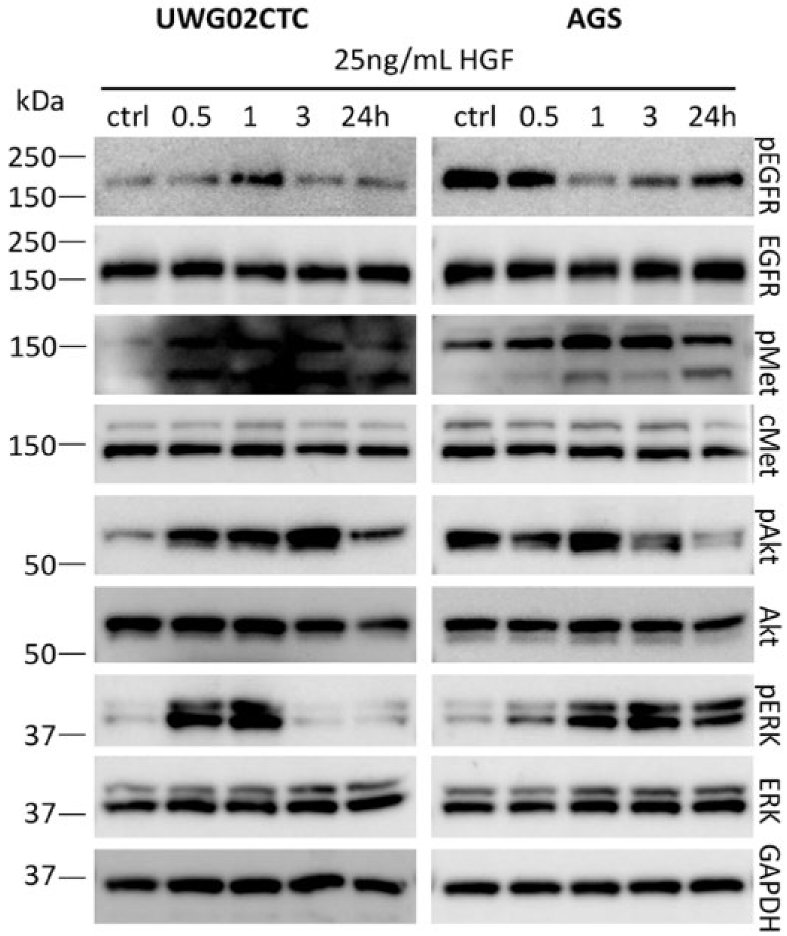

Supplementary Figure S1: Representative western blots showing total and phosphorylated protein levels in response to treatment with 25ng/mL with HGF for the times shown after serum starvation under 2D culture conditions (similar results found with 5 ng/mL HGF; data not shown). ctrl = control.

1 µM PIK-75

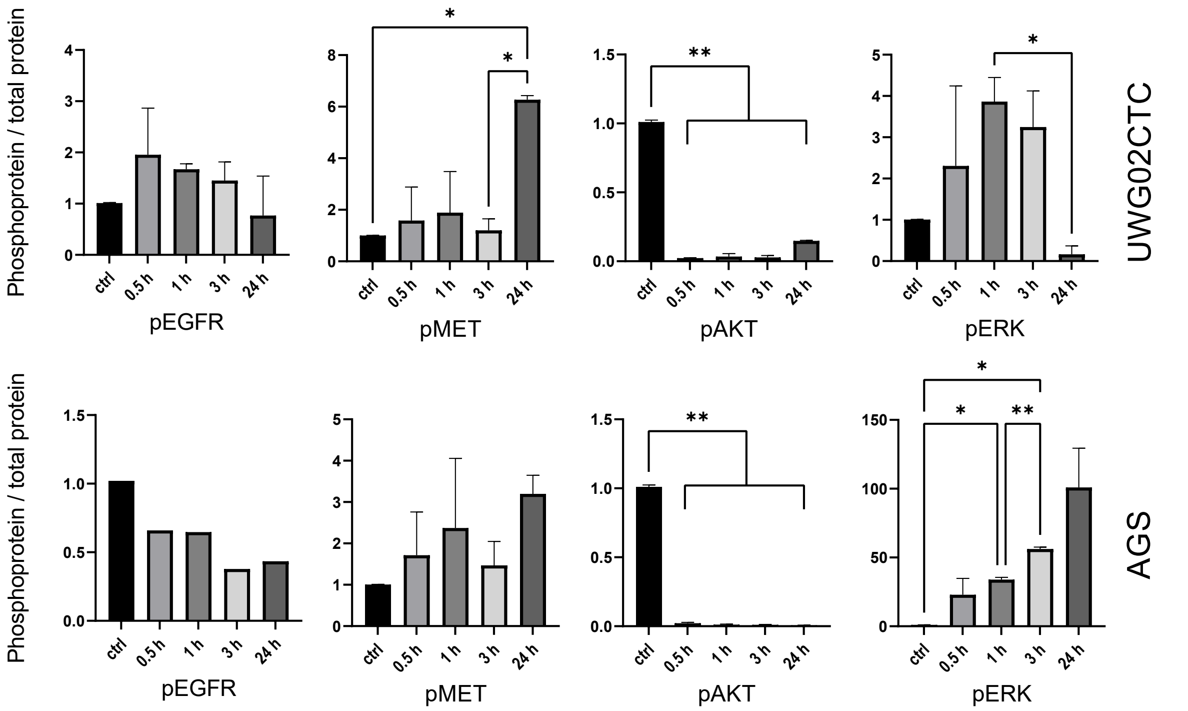

Supplementary Figure S2: Densitometry analysis of Western blots showing mean ± SEM, n = 2. Statistical significance was determined using Brown Forsythe ANOVA. $p < 0.05$; **, $p \leq 0.01$; ***, $p \leq 0.001$.

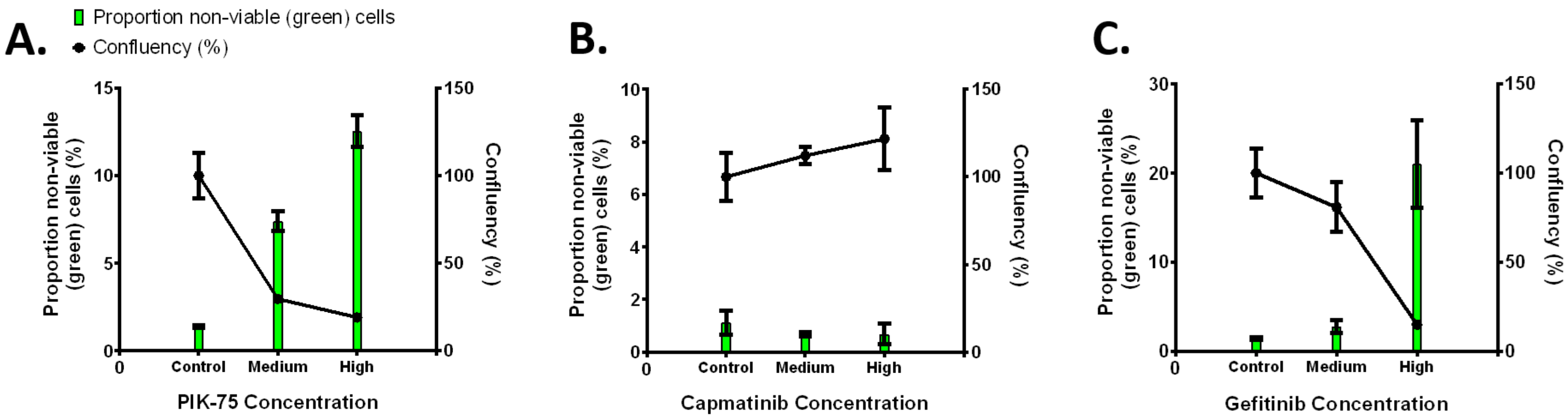

Supplementary Figure S3: Proportion of non-viable (stained with Cytotox Green) UWG02CTCs treated with **A)** PIK-75, **B)** capmatinib or **C)** gefitinib for 72 h, compared to overall confluency using data acquired from confluence mask analysis using IncuCyte Zoom software. 15,000 cells were seeded per well in 96 well plates and incubated at 37°C in hypoxic conditions for 24 h before the addition of drug. Cells treated with serial dilutions of drugs and 25 nM Cytotox Green reagent added to wells receiving high (1 µM PIK-75, 50 µM gefitinib, 5 µM capmatinib), intermediate (62.5nM PIK-75, 617nM gefitinib, 312nM capmatinib), and control doses of drug for visualisation of cell viability. Plates were imaged using IncuCyte ZOOM and confluence mask analysis performed to quantify development of green fluorescence (524 nm). Graphs show proportion of non-viable (green) cells, determined by dividing average green object area (μm^2) by average phase object area (μm^2), presented as columns, in addition to overall confluency, depicted as a line graph. Values shown are mean ± SEM and representative of at least two independent experiments.

250 nM GEFITINIB

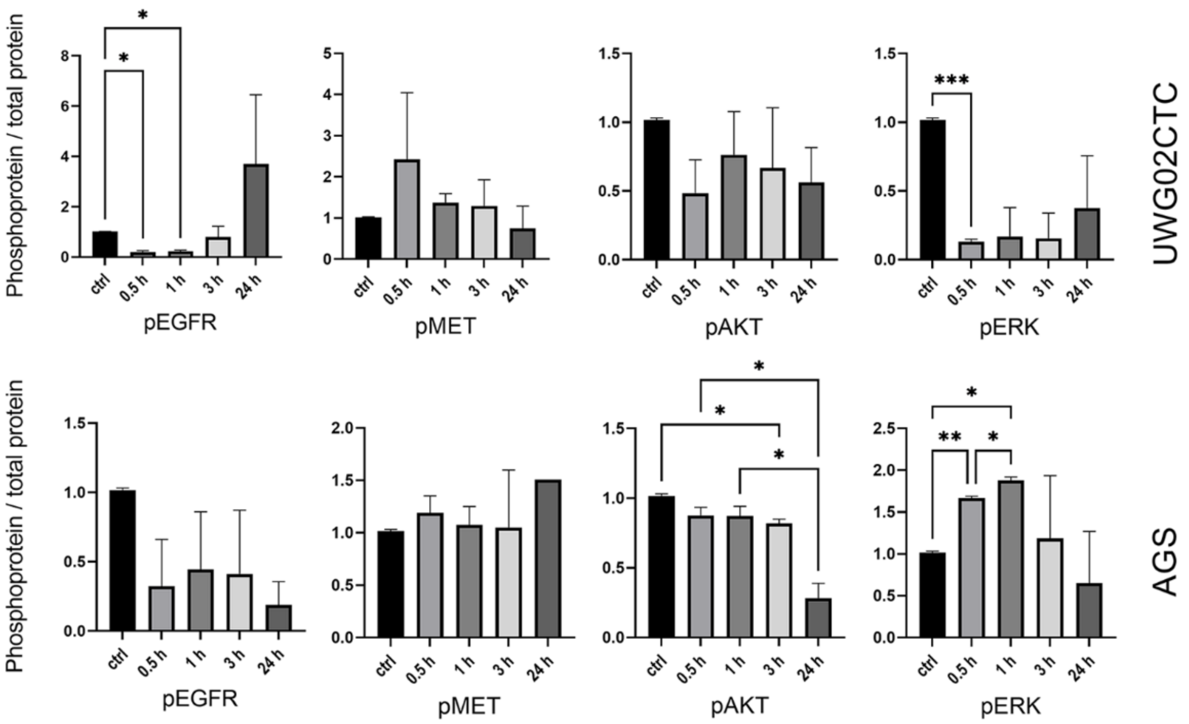

Supplementary Figure S4: Densitometry analysis of Western blots showing mean ± SEM. Statistical significance was determined using Brown Forsythe ANOVA. n = 3 for UWG02CTCs and n = 2 for AGS cells. p < 0.05; **, p ≤ 0.01; ***, p ≤ 0.001.

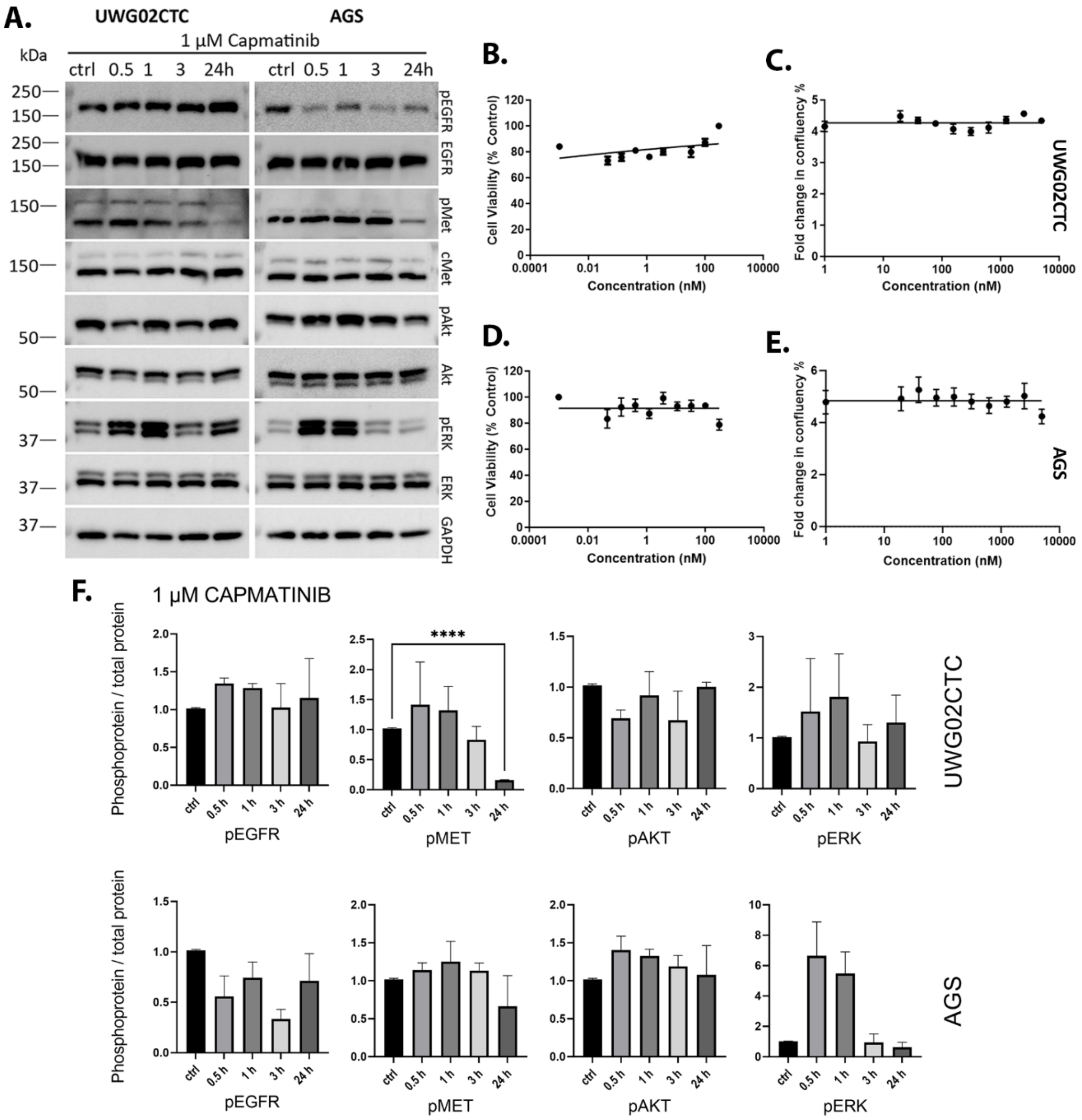

Supplementary Figure S5: A) Representative Western blots showing total and phosphorylated protein levels in response to pre-treatment with 1 µM capmatinib for the times shown for both cell lines under 2D complete growth media conditions. The housekeeping gene GAPDH was used as a total protein loading control. **B,D)** Representative UWG02CTC and AGS viability or **C,E)** confluency curves after treatment with increasing concentrations of capmatinib for 72 h under 2D complete growth media conditions. Values indicate mean cell viability or fold-change from seeding density in confluency normalised to control (no drug) ± SEM and are representative of three independent experiments. **F)** Densitometry analysis of western blots. Statistical significance was determined using Brown Foresythe ANOVA. $p < 0.05$; **, $p \leq 0.01$; ***, $p \leq 0.001$. Values shown are mean ± SEM. n = 2.

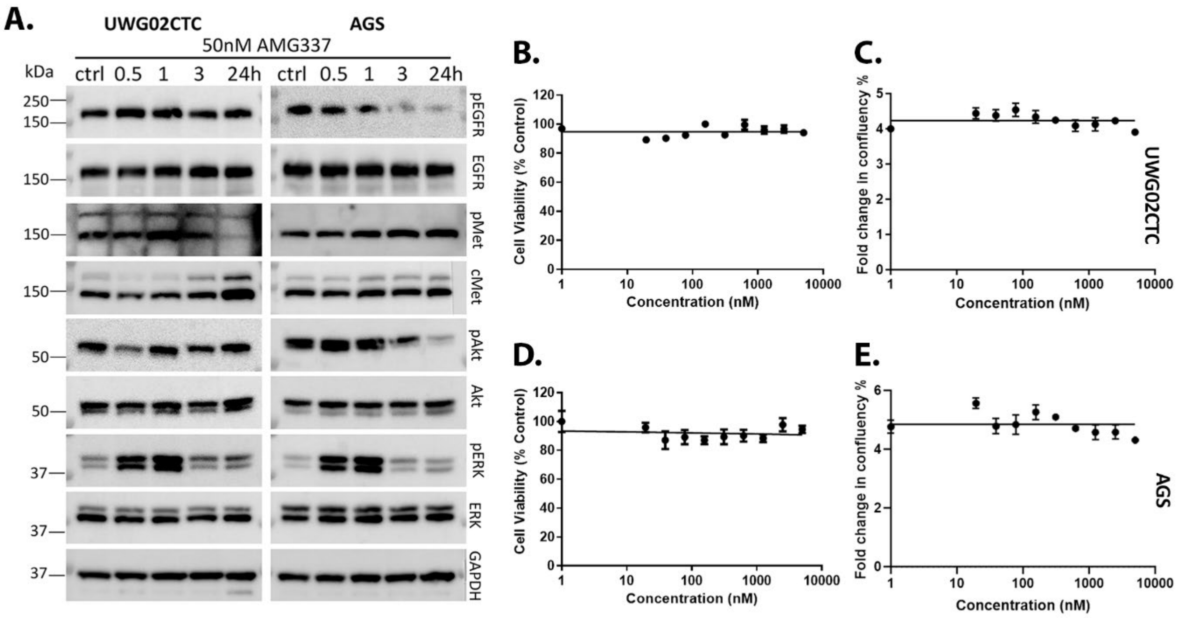

Supplementary Figure S6: A) Representative Western blots showing total and phosphorylated protein levels in response to pre-treatment with 50 nM AMG337 for the times shown for both cell lines under 2D complete growth media conditions. The housekeeping gene GAPDH was used as a total protein loading control. **B, D)** Representative UWG02CTC and AGS viability curves, or **C, E)** confluency curves after treatment with increasing concentrations of AMG337 for 72 h under 2D complete growth media conditions. Values indicate mean cell viability or fold-change from seeding density in confluency normalised to control (no drug) ± SEM and are representative of three independent experiments.

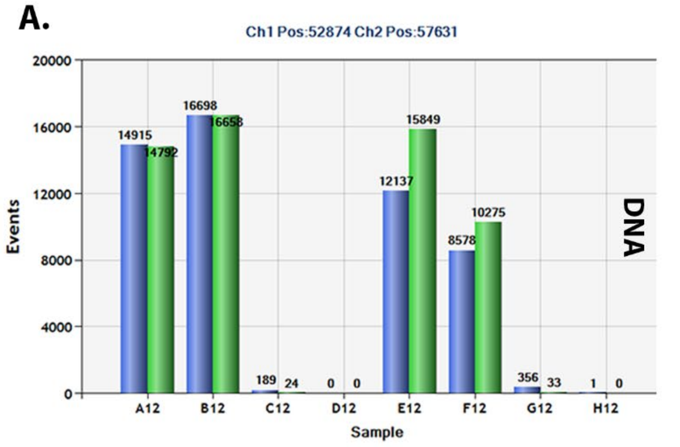

	AGS	CTC	H1993
c-MET x6/TBP	1	1	7.9
c-MET X20/TBP	0.76	0.83	10.8

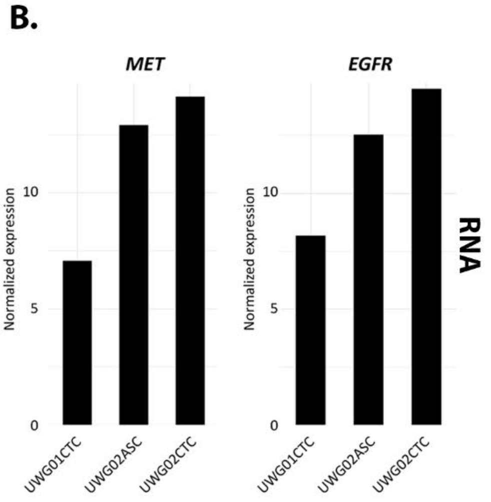

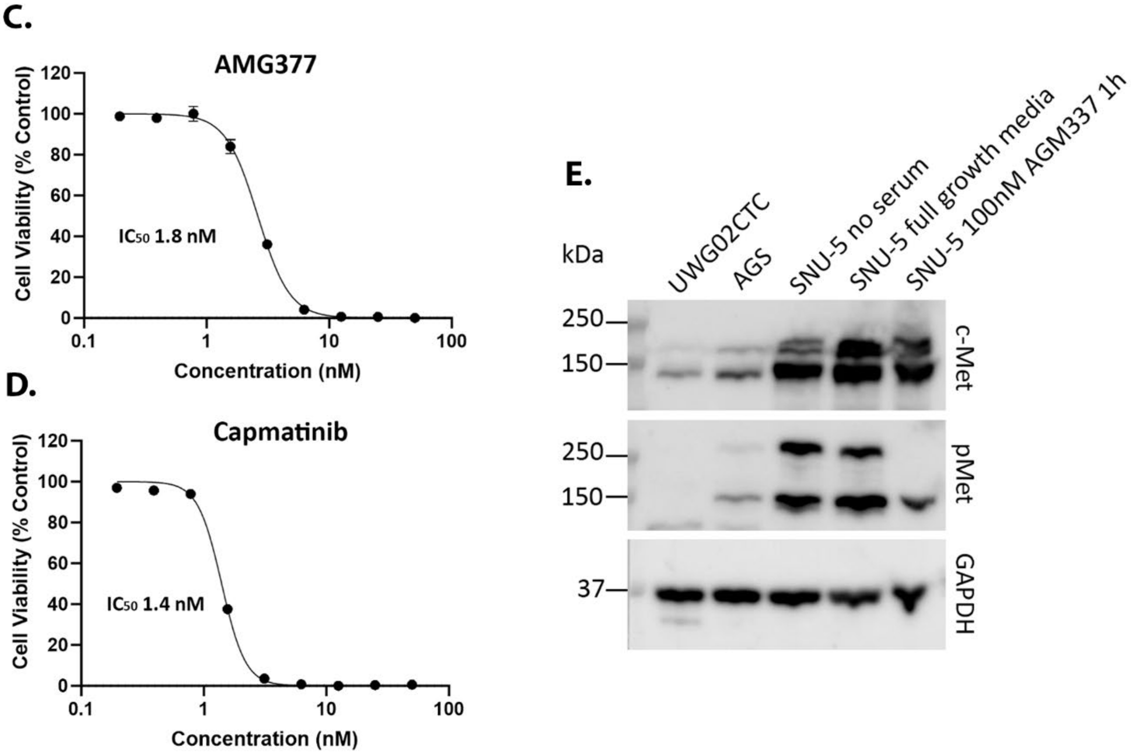

Supplementary Figure S7: A) Digital droplet PCR confirms that neither UWG02CTC nor AGS cells have *MET* amplification at the DNA level. **B)** RNA expression levels of *MET* and *EGFR* are relatively high in UWG02CTC and UWG02ASC cells compared to UWG01CTC (a CTC cell line derived from a patient with a metastatic neuroendocrine gastroesophageal cancer {Brungs, 2020 #4}. RNA sequencing performed using Illumina NovaSeq 6000. Data was normalised using DESeq2-normalisation by means ratio. **C, D)** Representative dose-response *MET*-amplified SNU-5 cell viability curves after treatment with AMG337 or capmatinib for 72 h. Values shown are mean ± SEM and are representative of two independent experiments. **E)** Western blot showing c-Met and pMet protein levels of UWG02CTC and AGS cells compared to SNU-5 cells in serum starved and full-growth media conditions in the absence of presence of 100nM AMG337 (pretreated for 1 h).

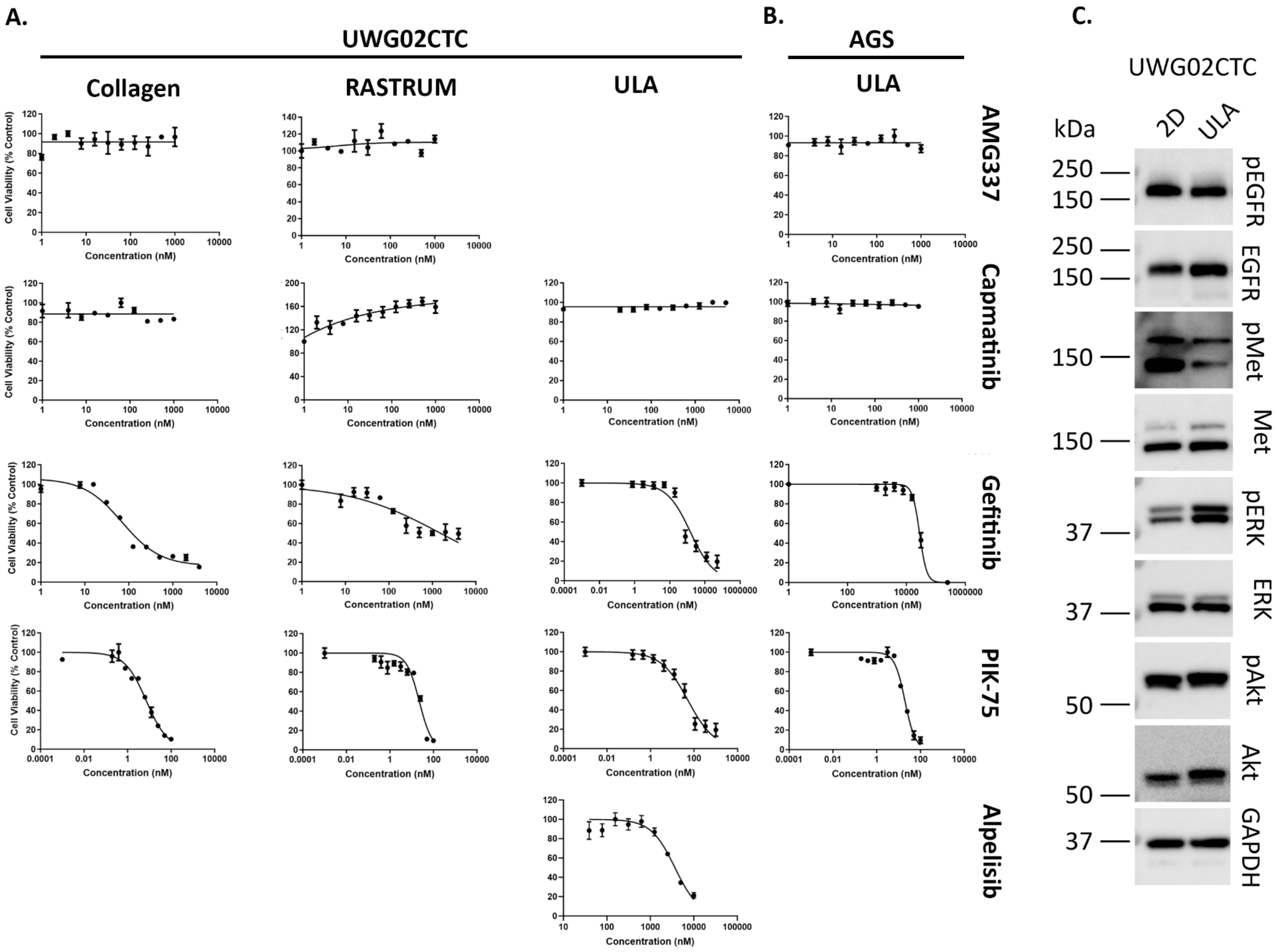
A.
UWG02CTC
Collagen
RASTRUM
ULA
B.
AGS
ULA
AMG337
Capmatinib
Gefitinib
PIK-75
Alpelisib
Cell Viability (% Control)
Concentration (nM)
C.
UWG02CTC
kDa
2D
ULA
250
150
pEGFR
EGFR
pMet
Met
37
pERK
ERK
50
pAkt
Akt
GAPDH

Supplementary Figure S8: Representative dose-response cell viability curves for all 3D cell culture conditions after treatment with PIK-75, alpelisib, gefitinib, AMG337 or capmatinib for 72 h. **A)** UWG02CTC and **B)** AGS cells. Values shown are mean ± SEM and are representative of at least two independent experiments. **C)** Western blot showing levels of total and phosphoproteins in UWG02CTC lysates from cells grown in 2D or ULA conditions with complete growth media.

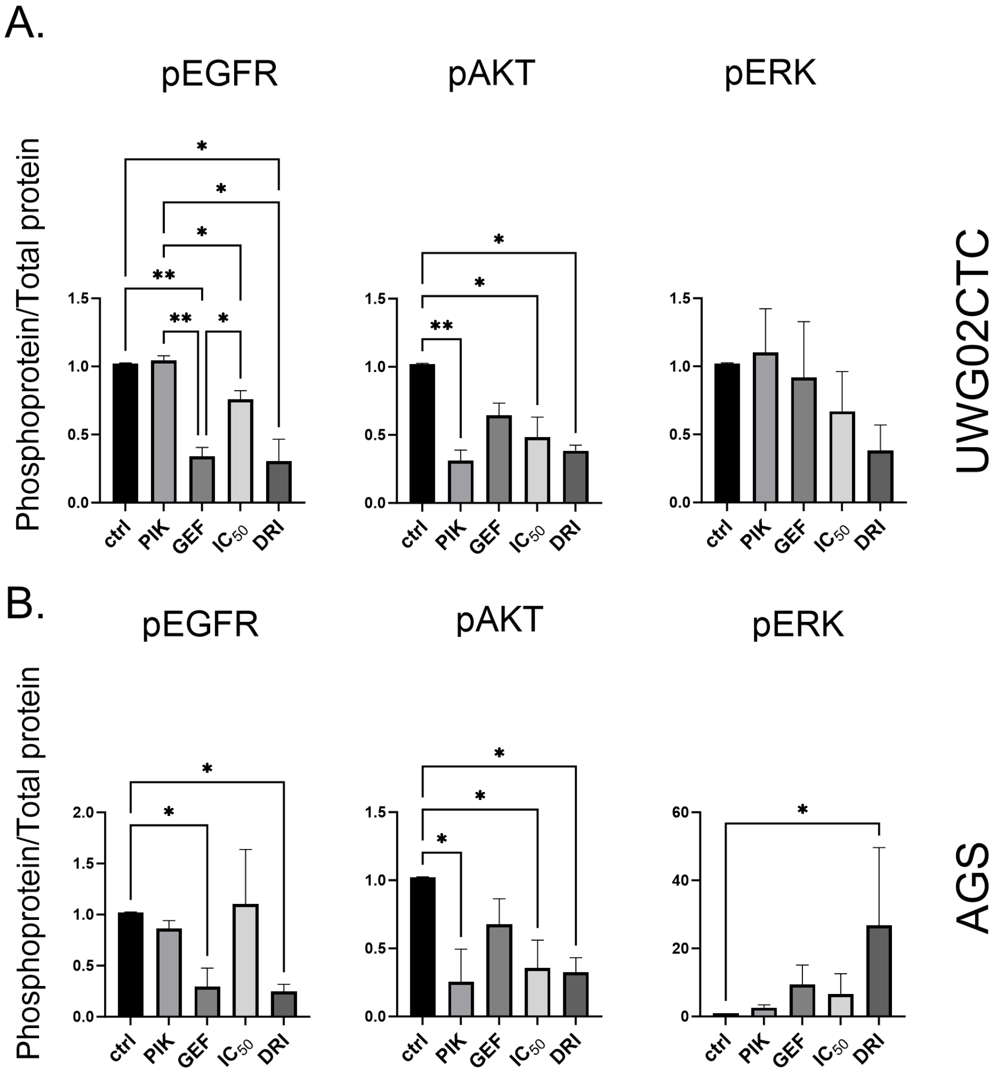

Supplementary Figure S9: Densitometry analysis of combination treatment Western blots. Cells were treated for 1 h with single agent or drug combinations at approximately either their IC_{50} concentrations derived from dose response curves as single agents or their (experimental or calculated) DRI concentrations for Fa = 0.5 derived from combination drug experiments under 2D conditions. For **A)** UWG02CTC this was 40 nM PIK-75 + 250 nM Gefitinib (IC_{50}) or 16 nM PIK-75 + 20 nM Gefitinib (DRI). For **B)** AGS this was 40 nM PIK-75 + 250 µM Gefitinib (IC_{50}); or 16 nM PIK-75 + 20 µM Gefitinib (DRI), n = 3. Statistical significance was determined using Brown Foresythe ANOVA. $p < 0.05$; **, $p \leq 0.01$; ***, $p \leq 0.001$. Values shown are mean ± SEM. n = 2

A.

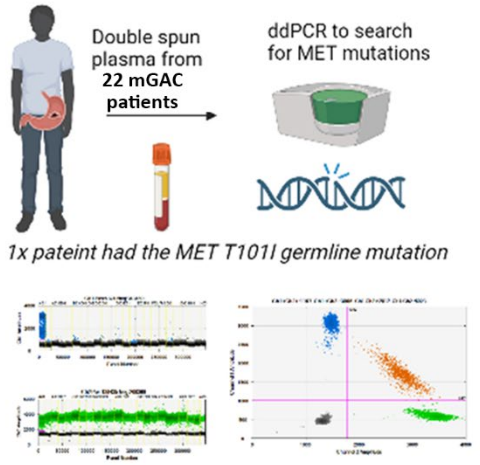

B.

Overall	
n	22
Gender = Male (%)	20 (90.9)
Age at diagnosis (mean (SD))	60.45 (16.02)
Histology type = ADENOCARCINOMA (%)	22 (100)
Surgical resection? (%)	6 (27.3)
Pre-treated	
Pre treated	7 (31.8)
Not resected	15 (68.2)
Co-morbidities (Total number) (mean (SD))	1.73 (1.93)

Location (%)	
Gastric	4 (21.1)
GOJ	7 (36.8)
Oesophagus	8 (42.1)
Recurrence/ Progression (%)	13 (59.1)
Dead at last contact (%)	19 (86.4)
Clinical Staging (%)	
IIB	1 (4.5)
III	5 (22.7)
IV	12 (54.5)
IVA	2 (9.1)
IVB	2 (9.1)

Supplementary Figure S10: A) Schematic of detection of germline *MET* T1010I mutation in 1/22 patients with metastatic gastric adenocarcinoma detected in cDNA from double-spun plasma via digital droplet PCR. **B)** Table with statistics of the 22 patients from this cohort. This was performed under a waiver of consent approved by the South West Sydney Local Health District human research ethic committee (No: HREC/15/LPOOL/121).
